# Acetaldehyde and defective mismatch repair increase colonic tumours in a Lynch syndrome model with *Aldh1b1* inactivation

**DOI:** 10.1242/dmm.050240

**Published:** 2023-07-31

**Authors:** Guia Cerretelli, Ying Zhou, Mike F. Müller, David J. Adams, Mark J. Arends

**Affiliations:** ^1^ University of Edinburgh, Division of Pathology, Centre for Comparative Pathology, CRUK Edinburgh Centre, Institute of Genetics and Cancer, Western General Hospital, Crewe Road South, Edinburgh EH4 2XR, UK; ^2^ Wellcome Sanger Institute, Hinxton, Cambridge CB10 1HH, UK

**Keywords:** Lynch syndrome, Mismatch repair, *Msh2*, ALDH1B1, Ethanol, Acetaldehyde, Colorectal cancer, Adenoma

## Abstract

ALDH1B1 expressed in the intestinal epithelium metabolises acetaldehyde to acetate, protecting against acetaldehyde-induced DNA damage. MSH2 is a key component of the DNA mismatch repair (MMR) pathway involved in Lynch syndrome (LS)-associated colorectal cancers. Here, we show that defective MMR (dMMR) interacts with acetaldehyde, in a gene/environment interaction, enhancing dMMR-driven colonic tumour formation in a LS murine model of *Msh2* conditional inactivation (*Lgr5-CreER*; *Msh2^flox/−^*, or Msh2-LS) combined with *Aldh1b1* inactivation. Conditional (*Aldh1b1^flox/flox^*) or constitutive (*Aldh1b1^−/−^*) *Aldh1b1* knockout alleles combined with the conditional *Msh2^flox/−^* intestinal knockout mouse model of LS (Msh2-LS) received either ethanol, which is metabolised to acetaldehyde, or water. We demonstrated that 41.7% of ethanol-treated *Aldh1b1^flox/flox^* Msh2-LS mice and 66.7% of *Aldh1b1^−/−^* Msh2-LS mice developed colonic epithelial hyperproliferation and adenoma formation, in 4.5 and 6 months, respectively, significantly greater than 0% in water-treated control mice. Significantly higher numbers of dMMR colonic crypt foci precursors and increased plasma acetaldehyde levels were observed in ethanol-treated *Aldh1b1^flox/flox^* Msh2-LS and *Aldh1b1^−/−^* Msh2-LS mice compared with those in water-treated controls. Hence, ALDH1B1 loss increases acetaldehyde levels and DNA damage that interacts with dMMR to accelerate colonic, but not small intestinal, tumour formation.

## Teaser

**Editors' choice:** Increased acetaldehyde levels are associated with acceleration of defective mismatch repair-driven colonic tumour development in models of Lynch syndrome with inactivated *Aldh1b1*, involving DNA damage responses and decreased apoptosis.

## INTRODUCTION

Constitutional (mostly germline heterozygous) pathogenic variants in one of the four DNA mismatch repair (MMR) genes (*MSH2*, *MLH1*, *MSH6* and *PMS2*) cause Lynch syndrome (LS), although, rarely, LS may be caused by certain mutations of *EPCAM* (immediately adjacent to *MSH2*) that also inactivate *MSH2* (Poulogiannis et al., 2010; Bellizzi and Frankel, 2009; Frankel et al., 2019). LS patients have an increased lifetime risk for several cancer types, mainly in the large intestine (LI) and endometrium, but also in the small intestine (SI), stomach, hepatobiliary tract, pancreas, skin (sebaceous tumours) and several other organs (Poulogiannis et al., 2010; Bellizzi and Frankel, 2009; Frankel et al., 2019). Variable expression of cancer predisposition phenotypes amongst LS patients suggests important effects of allelic variation, genetic modifiers, sex differences and lifestyle and/or environmental factors, together with complex genetic and environmental interactions (van Duijnhoven et al., 2013). Therefore, it is important to identify environmental risk factors and quantify their risks for developing cancer for LS patients in order to provide guidance for cancer surveillance and care, as well as understanding the underlying biology (The Prospective Lynch Syndrome Database, https://www.ehtg.org/plsd.php; Møller et al., 2017).

Chronic high alcohol consumption is associated with cancers of the liver, breast, upper aerodigestive tract and bowel, and ethanol has been declared a group 1 carcinogen by the International Agency for Research on Cancer (IARC) (IARC, 2010). There have been very few studies on whether alcohol affects colorectal cancer risk in LS patients, apart from a cross-sectional multicentre study that showed that alcohol consumption is significantly correlated with increased risk of early-onset colorectal cancer in LS patients that were carriers of pathogenic *MLH1* or *MSH2* variants, with tumours located in the proximal colon (Miguchi et al., 2018; Fujiyoshi et al., 2022). The potential mechanisms of how ethanol contributes to intestinal carcinogenesis include involvement of its metabolite acetaldehyde. Acetaldehyde is a highly reactive small aldehyde capable of inducing a wide range of DNA damage (IARC, 2010; Stagos et al., 2010). Evidence for acetaldehyde involvement in ethanol-related cancers emerged from the study of human polymorphic variants in alcohol dehydrogenases and aldehyde dehydrogenases. A polymorphism of ALDH1B1 causes reduced enzyme activity (Jackson et al., 2015) and has been associated with altered drinking habits and alcohol sensitivity in people of European origin (Husemoen et al., 2008; Linneberg et al., 2010). ALDH1B1 plays a key role in acetaldehyde detoxification to acetate in the intestinal epithelium (Stagos et al., 2010; Husemoen et al., 2008). We previously demonstrated an important role of murine ALDH1B1 in acetaldehyde detoxification *in vivo* during intestinal tumorigenesis in wild-type (wt) and ALDH1B1-depleted mice after long-term ethanol treatment for 1 year (Müller et al., 2016; Skarnes et al., 2011). We also showed that ethanol can accelerate colonic tumour formation in a mouse model of LS (*Lgr5-CreER*; *Msh2^flox/−^*), known as Msh2-LS, involving scattered foci of intestinal conditional inactivation of MSH2 function (by tamoxifen-induced transient Cre activation) in LGR5-expressing intestinal epithelial stem cells (Barker et al., 2007; Claij and te Riele, 2004; de Wind et al., 1995; Wojciechowicz et al., 2014; Cerretelli et al., 2021). In this work, we aimed to study the combined effects of inactivation of *Msh2* and *Aldh1b1* in mice with long-term ethanol treatment using this Msh2-LS mouse model (Wojciechowicz et al., 2014; Cerretelli et al., 2021; Dole and Gentry, 1984; Holmes et al., 1986), in which we introduced by cross-breeding either the conditional inactivation *Aldh1b1* allele (*Aldh1b1^flox/flox^*) or the constitutive knockout *Aldh1b1* allele (*Aldh1b1^−/−^*) (Müller et al., 2016; Skarnes et al., 2011).

In *Aldh1b1^flox/flox^* Msh2-LS mice, the conditional loss of ALDH1B1 and MSH2 expression occurs in scattered LGR5-expressing SI and LI epithelial stem cells. We hypothesised that LGR5-expressing intestinal epithelial cells and their daughter cells would acquire more DNA mutations owing to the combined lack of MMR pathway activity and elevated levels of acetaldehyde-mediated DNA damage. In addition, *Aldh1b1^−/−^* Msh2-LS mice are characterised by complete loss of ALDH1B1 expression in all cells of the organism. We hypothesised that widespread loss of ALDH1B1 further increases acetaldehyde levels, causing more acetaldehyde-mediated DNA damage, which may interact with defective MMR to enhance intestinal tumour formation.

## RESULTS

### Acetaldehyde causes increased colonic tumour development in *Aldh1b1^flox/flox^* Msh2-LS mice and *Aldh1b1^−/−^* Msh2-LS mice

To assess whether intestinal tumour formation is affected by acetaldehyde levels in Msh2-LS mice with either conditional or constitutive inactivation of *Aldh1b1*, mice were induced with tamoxifen (causing transient Cre activation in scattered LGR5-expressing intestinal epithelial stem cells) to conditionally inactivate *Msh2*. Subsequently, the mice were treated with either 20% ethanol or water to drink over the long-term and were observed for up to 12 months for intestinal tumour formation.

**Fig. 1.**
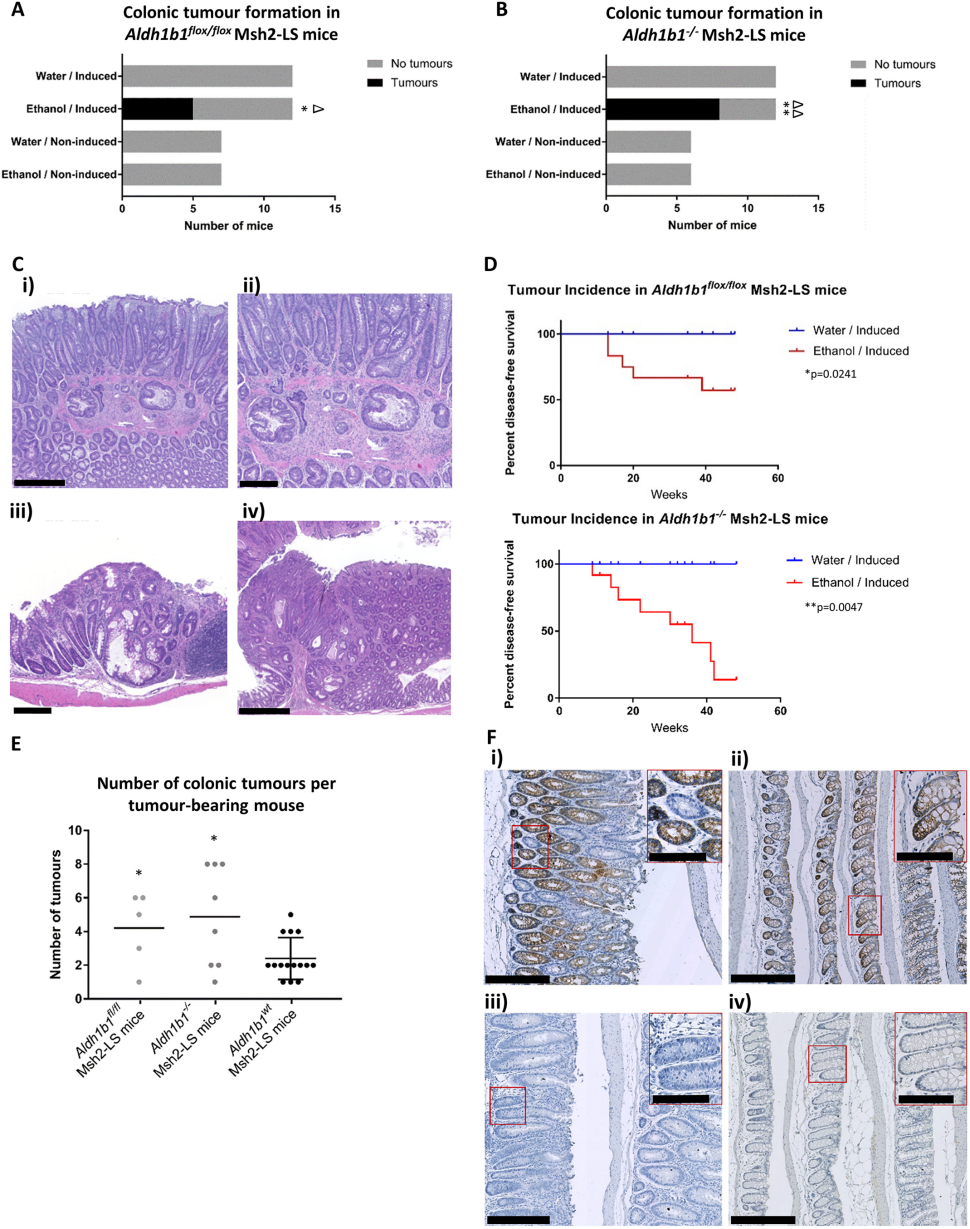
**Colorectal tumour formation (adenomas and adenocarcinomas) in *Aldh1b1^flox/flox^* and *Aldh1b1^−/−^* Msh2-LS mice after receiving 20% ethanol or water.** (A) Bar chart of the numbers of *Aldh1b1^flox/flox^* Msh2-LS mice that developed large intestinal tumours: 5/12 (41.7%) ethanol-treated induced *Aldh1b1^flox/flox^* Msh2-LS mice developed large intestinal tumours compared with 0/12 (0%) water-treated induced *Aldh1b1^flox/flox^* Msh2-LS mice (Fisher's exact test, *P=0.0373). In both groups of non-induced *Aldh1b1^flox/flox^* Msh2-LS mice (water-treated and ethanol-treated), 0/7 (0%) non-induced *Aldh1b1^flox/flox^* Msh2-LS control mice developed colonic neoplasms (Fisher's exact test, no significant differences observed). Comparison of the tumour-bearing ethanol-treated tamoxifen-induced *Aldh1b1^flox/flox^* Msh2-LS mice that developed colonic tumours with ethanol-treated non-induced *Aldh1b1^flox/flox^* Msh2-LS mice that developed no large intestinal tumours showed a significant difference (Fisher's exact test, ^Δ^*P*=0.0466). (B) Bar chart of the numbers of *Aldh1b1*^−/−^ Msh2-LS mice that developed large intestinal tumours: 8/12 (66.7%) ethanol-treated induced *Aldh1b1^−/−^* Msh2-LS mice developed large intestinal tumours compared with 0/12 (0%) water-treated induced *Aldh1b1^−/−^* Msh2-LS mice (Fisher's exact test, **P=0.0013). In both groups of non-induced *Aldh1b1^−/−^* Msh2-LS mice (water-treated and ethanol-treated), 0/6 (0%) non-induced *Aldh1b1^−/−^* Msh2-LS control mice developed colonic neoplasms (Fisher's exact test, no significant differences observed). Comparison of the tumour-bearing ethanol-treated induced *Aldh1b1^−/−^* Msh2-LS mice with ethanol-treated non-induced *Aldh1b1^−/−^* Msh2-LS mice that developed no large intestinal tumours showed a significant difference (Fisher's exact test, ^ΔΔ^*P*=0.0073). (C) Large intestinal tumour histology. Representative histological images of a colonic adenocarcinoma showing invasion through the muscularis mucosae into the submucosa (i,ii); a proximal colonic adenoma (iii) from ethanol-treated induced *Aldh1b1^flox/flox^* Msh2-LS mice; and a proximal colonic adenoma from an ethanol-treated induced *Aldh1b1^−/−^* Msh2-LS mouse (iv). (D) Tumour incidence shown as survival curves in tamoxifen-induced *Aldh1b1^flox/flox^* (top) or *Aldh1b1^−/−^* (bottom) Msh2-LS mice treated with either 20% ethanol (red) or water (blue) [log-rank (Mantel–Cox) test, *P=0.0241 (top), **P=0.0047 (bottom)]. (E) Plot of the number of tumours per tumour-bearing mouse in ethanol-treated *Aldh1b1^flox/flox^* (average 4.2), *Aldh1b1^−/−^* (average 4.8) and *Aldh1b1^wt^* (average 2.4) (previous data, Cerretelli et al., 2021) Msh2-LS tumour-bearing mice. Unpaired two-tailed Student's *t*-test showed statistically significant differences for comparisons of *Aldh1b1^flox/flox^* Msh2-LS tumour-bearing mice versus *Aldh1b1^wt^* Msh2-LS tumour-bearing mice (*P=0.0319), and for *Aldh1b1^−/−^* Msh2-LS tumour-bearing mice versus *Aldh1b1^wt^* Msh2-LS tumour-bearing mice (*P=0.0103), but no statistically significant difference was observed between *Aldh1b1^flox/flox^* Msh2-LS tumour-bearing mice and *Aldh1b1^−/−^* Msh2-LS tumour-bearing mice. (F) Representative images of ALDH1B1 immunostaining of murine colonic crypt epithelium with some ALDH1B1-negative crypts in ethanol-treated induced *Aldh1b1^flox/flox^* Msh2-LS mice (i), ALDH1B1 immunostaining of murine colonic crypt epithelium with all crypts positive for ALDH1B1 in ethanol-treated non-induced *Aldh1b1^flox/flox^* Msh2-LS mice (ii), ALDH1B1 immunostaining of murine colonic crypt epithelium with all crypts lacking ALDH1B1 expression in ethanol-treated induced *Aldh1b1^−/−^* Msh2-LS mice (iii) and ethanol-treated non-induced *Aldh1b1^−/−^* Msh2-LS mice (iv). Images are representative of *n*=4-7 mice per group. Scale bars: 250 µm (Ci,Civ, Fi-iv); 100 µm (Cii,Ciii; insets in Fi-iv).

Following ethanol treatment, most of the induced *Aldh1b1^flox/flox^* Msh2-LS and *Aldh1b1^−/−^* Msh2-LS mice displayed either anal prolapse or >20% reduction in body weight and were culled for necropsy and tissue collection (Kilkenny et al., 2010). In the ethanol-treated induced *Aldh1b1^flox/flox^* Msh2-LS mice, 5/12 (41.6%) demonstrated colonic epithelial hyperproliferation with colonic adenoma formation and, in one case, invasive adenocarcinoma, all within an average of 4.5 months (Fig. 1A,C; Fig. S1A-D). One ethanol-treated induced *Aldh1b1^flox/flox^* Msh2-LS mouse showed only colonic hyperproliferation without tumour formation. Invasive adenocarcinoma was observed developing from a colonic adenoma in one (8.4%) of the 12 ethanol-treated induced *Aldh1b1^flox/flox^* Msh2-LS mice (20% of adenoma-bearing mice) (Fig. 1C). Seven of 12 ethanol-treated induced *Aldh1b1^flox/flox^* Msh2-LS mice did not show any intestinal tumour formation, but a uterine endometrial adenocarcinoma was found in one mouse after 13 weeks of ethanol treatment (Fig. S2) – a tumour type frequently seen in LS. No SI tumours or SI hyperproliferative zones were observed in any of these mice (Fig. S1E-H). No small intestinal or colonic abnormalities (adenomas, adenocarcinomas or hyperproliferation) were observed in any of the 12 water-treated induced *Aldh1b1^flox/flox^* Msh2-LS mice (Fig. 1A). In the ethanol-treated non-induced *Aldh1b1^flox/flox^* Msh2-LS mice, 3/7 (43%) showed zones of colonic crypt epithelial hyperproliferation, involving mostly the proximal and mid-colon (Fig. 1A; Fig. S1). None of the ethanol-treated non-induced *Aldh1b1^flox/flox^* Msh2-LS control mice showed intestinal adenoma or adenocarcinoma formation. In the water-treated non-induced *Aldh1b1^flox/flox^* Msh2-LS mice, no intestinal hyperproliferation, adenoma/adenocarcinoma or other abnormalities were observed (Fig. 1A). The incidence of colonic adenoma/adenocarcinoma was statistically significantly greater in ethanol-treated induced *Aldh1b1^flox/flox^* Msh2-LS mice (*P*=0.0373, Fisher's exact test; *P*=0.0241, Mantel–Cox test for survival difference) (Fig. 1D) compared with that in water-treated induced *Aldh1b1^flox/flox^* Msh2-LS mice, and also compared with that in ethanol-treated (and water-treated) non-induced *Aldh1b1^flox/flox^* Msh2-LS control mice (*P*=0.0466 for both comparisons, Fisher's exact test), but no significant differences were observed between ethanol-treated and water-treated non-induced *Aldh1b1^flox/flox^* Msh2-LS control mice (Fig. 1A).

In the ethanol-treated induced *Aldh1b1^−/−^* Msh2-LS mice, 8/12 (66.7%) demonstrated large intestinal epithelial hyperproliferation with colonic adenoma formation, all within an average of 6 months (Fig. 1B; Fig. S3A-D), but no adenocarcinomas were observed. Four of 12 ethanol-treated induced *Aldh1b1^−/−^* Msh2-LS mice did not show any intestinal tumour formation. No SI tumours or SI hyperproliferative zones were observed in any of these mice (Fig. S3E-H). No SI or LI abnormalities were observed in any of the 12 water-treated induced *Aldh1b1^−/−^* Msh2-LS mice (Fig. 1B).

In the ethanol-treated non-induced *Aldh1b1^−/−^* Msh2-LS mice, 2/6 (33.4%) showed zones of colonic crypt epithelial hyperproliferation (mainly in the proximal and mid-colon) (Fig. S3). None of the ethanol-treated and water-treated non-induced *Aldh1b1^−/−^* Msh2-LS mice showed intestinal adenoma/adenocarcinoma formation (Fig. 1B). The incidence of colonic adenoma was statistically significantly greater in ethanol-treated induced *Aldh1b1^−/−^* Msh2-LS mice (*P*=0.0013, Fisher's exact test; *P*=0.0047, Mantel–Cox test for survival difference) (Fig. 1D) compared with that in water-treated induced *Aldh1b1^−/−^* Msh2-LS mice, and also compared with that in ethanol-treated (and water-treated) non-induced *Aldh1b1^−/−^* Msh2-LS control mice (*P*=0.0073 for both comparisons, Fisher's exact test) (Fig. 1B). No differences were observed between ethanol-treated and water-treated non-induced *Aldh1b1^−/−^* Msh2-LS mice (Fig. 1B). Although the patterns of tumour distribution were similar, the numbers of tumours per tumour-bearing mouse were statistically significantly greater in both ethanol-treated induced *Aldh1b1^flox/flox^* Msh2-LS and ethanol-treated induced *Aldh1b1^−/−^* Msh2-LS mice compared with those in ethanol-treated induced *Aldh1b1^wt^* Msh2-LS mice (Fig. 1E; Fig. S4A-D) (previous data from Cerretelli et al., 2021). No tumours were observed in the SI, stomach, liver, hepato-biliary tract, pancreas, spleen, lymph nodes or thymus in any of the induced or non-induced, water-treated or ethanol-treated *Aldh1b1^flox/flox^* Msh2-LS and *Aldh1b1^−/−^* Msh2-LS mice.

### Loss of expression of MSH2 and ALDH1B1 proteins in *Aldh1b1^flox/flox^* and *Aldh1b1^−/−^* Msh2-LS mice

**Fig. 2.**
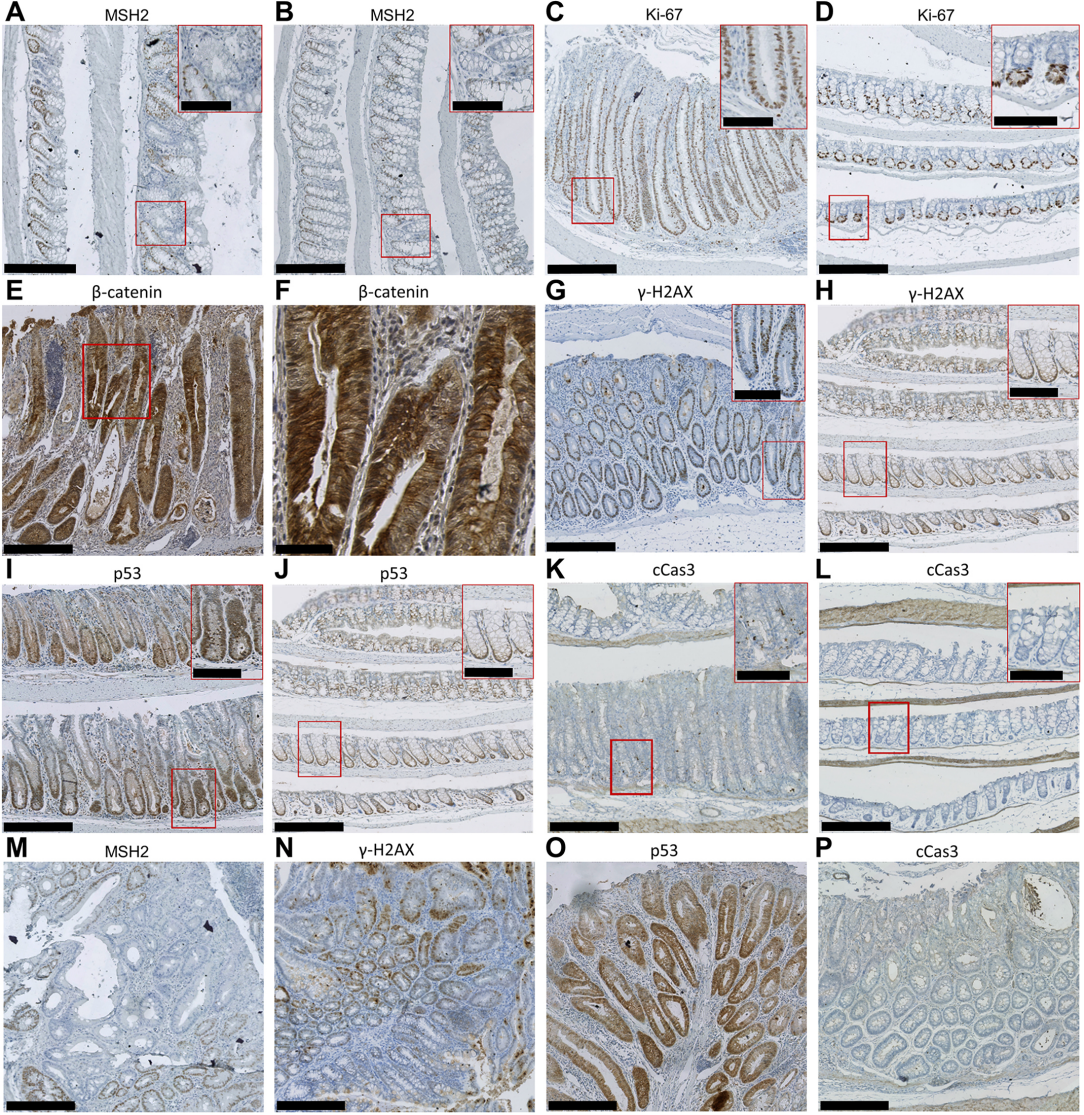
**Representative histological images of IHC analysis of induced *Aldh1b1^−/−^* Msh2-LS murine colonic mucosal epithelium and adenomas.** (A,B) MSH2 immunostaining of the murine colon showing some MSH2-negative crypts from ethanol-treated (A) and water-treated (B) induced *Aldh1b1*^−/−^ Msh2-LS mice. (C,D) Ki-67 immunostaining of the murine colon from ethanol-treated induced *Aldh1b1^−/−^* Msh2-LS mice showing hyperproliferative crypt elongation (C) and water-treated induced *Aldh1b1^−/−^* Msh2-LS mice with normal length crypts (D). (E,F) β-catenin immunostaining in a colonic adenoma from an ethanol-treated induced *Aldh1b1*^−/−^ Msh2-LS mouse (E), with the selected area (red rectangle) within E magnified in F. (G,H) γ-H2AX immunostaining in ethanol-treated (G) and water-treated (H) induced *Aldh1b1^−/−^* Msh2-LS mice. (I,J) p53 immunostaining in ethanol-treated (I) and water-treated (J) induced *Aldh1b1^−/−^* Msh2-LS murine colons. (K,L) cCas3 immunostaining in ethanol-treated (K) and water-treated (L) induced *Aldh1b1^−/−^* Msh2-LS murine colons. (M-P) Representative images of colonic adenomas from ethanol-treated induced *Aldh1b1^−/−^* Msh2-LS mice immunostained for MSH2 (M), γ-H2AX (N), p53 (O) and cCas3 (P). Images are representative of *n*=4-7 mice per group. Scale bars: 250 µm (A-E,G-P); 50 µm (F); 100 µm (insets in A-D,G-L).

**Fig. 3.**
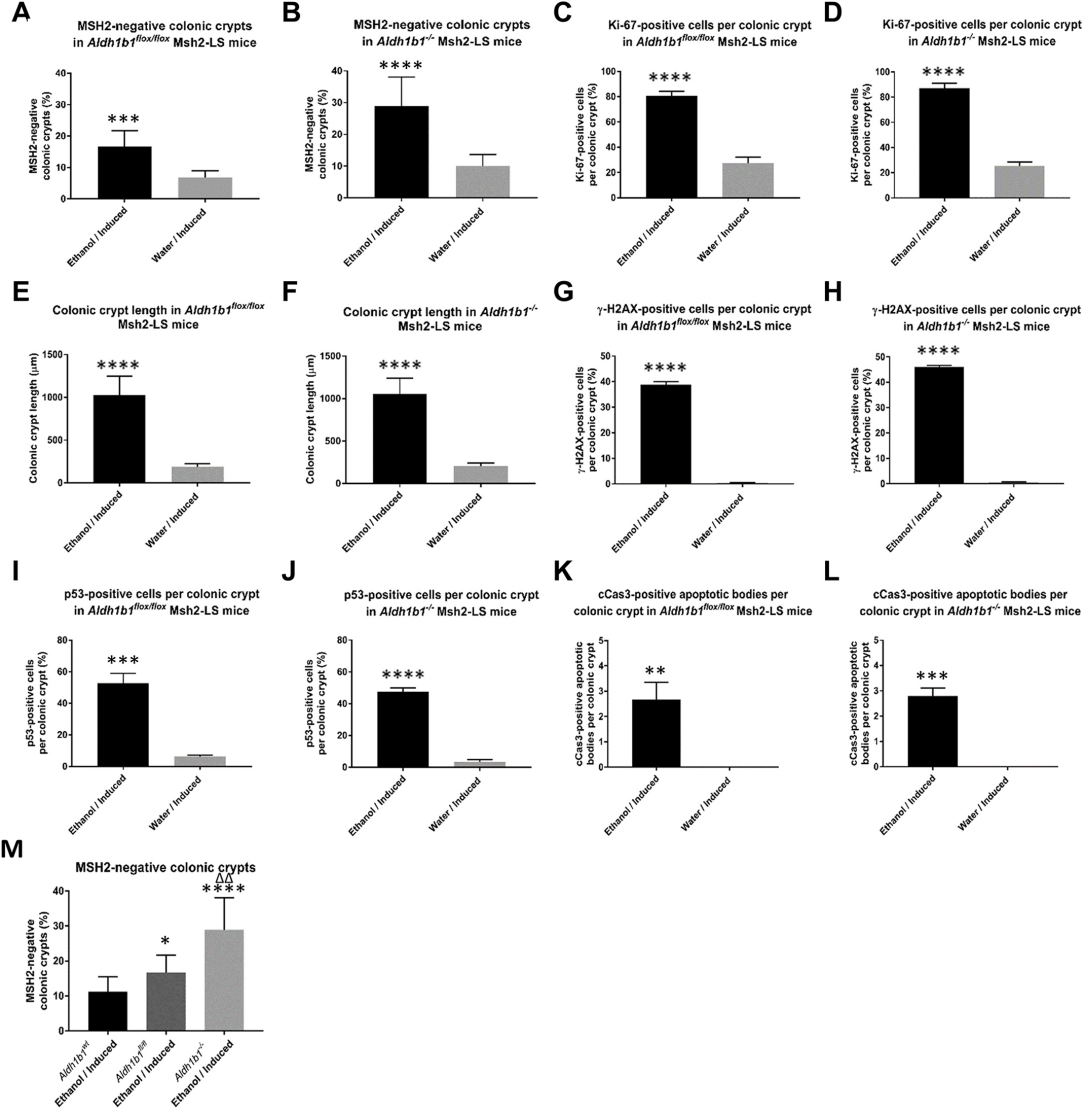
**Quantitative analyses of immunohistochemical stains of murine colonic mucosa from ethanol-treated and water-treated induced *Aldh1b1^flox/flox^* and *Aldh1b1^−/−^* Msh2-LS mice.** (A,B) Percentages of MSH2-negative colonic crypts in induced *Aldh1b1^flox/flox^* (A) and *Aldh1b1^−/−^* (B) Msh2-LS mice [unpaired two-tailed Student's *t*-test, ***P=0.0006 (A) and ****P<0.0001 (B) versus water]. (C,D) Percentages of Ki-67-positive cells per colonic crypt in induced *Aldh1b1^flox/flox^* (C) and *Aldh1b1^−/−^* (D) Msh2-LS mice (unpaired two-tailed Student's *t*-test, ****P<0.0001 versus water). (E,F) Colonic crypt lengths (μm) in induced *Aldh1b1^flox/flox^* (E) and *Aldh1b1^−/−^* (F) Msh2-LS mice (Mann–Whitney *U*-test, ****P<0.0001 versus water). (G,H) Percentages of γ-H2AX-positive cells per colonic crypt in induced *Aldh1b1^flox/flox^* (G) and *Aldh1b1^−/−^* (H) Msh2-LS mice (unpaired two-tailed Student's *t*-test, ****P<0.0001 versus water). (I,J) Percentages of p53-positive cells per colonic crypt in induced *Aldh1b1^flox/flox^* (I) and *Aldh1b1^−/−^* (J) Msh2-LS mice [unpaired two-tailed Student's *t*-test, ***P<0.0002 (I) and ****P<0.0001 (J) versus water]. (K,L) Number of cleaved Caspase-3 (cCas3)-positive apoptotic bodies per colonic crypt in induced *Aldh1b1^flox/flox^* (K) and *Aldh1b1^−/−^* (L) Msh2-LS mice [unpaired two-tailed Student's *t*-test, **P=0.0024 (K) and ***P=0.0001 (L) versus water]. Data are shown as mean±s.d., *n*=6 for all comparisons. (M) Comparison of the percentage of MSH2-negative crypts in colonic mucosa of ethanol-treated induced *Aldh1b1^wt^* Msh2-LS mice (previous data, Cerretelli et al., 2021), *Aldh1b1^flox/flox^* Msh2-LS mice and *Aldh1b1^−/−^* Msh2-LS mice. Unpaired two-tailed Student's *t*-test showed statistically significant differences for comparisons of *Aldh1b1^flox/flox^* Msh2-LS mice versus *Aldh1b1^wt^* Msh2-LS mice (*P=0.0262), for *Aldh1b1^−/−^* Msh2-LS versus *Aldh1b1^wt^* Msh2-LS mice (****P<0.0001), and for *Aldh1b1^−/−^* Msh2-LS mice versus *Aldh1b1^flox/flox^* Msh2-LS mice (^ΔΔ^*P*=0.0040).

Immunostaining for MSH2 showed that all colonic adenomas tested from both ethanol-treated induced *Aldh1b1^−/−^* Msh2-LS and induced *Aldh1b1^flox/flox^* Msh2-LS mice had MSH2-negative dysplastic glands, surrounded by or admixed with MSH2-positive crypts showing normal, reactive or hyperproliferative changes (Fig. 2A,B,M; Fig. S5A,B,M). The percentage of MSH2-negative colonic crypts in the entire colon was statistically significantly higher in ethanol-treated induced *Aldh1b1^−/−^* Msh2-LS mice compared with that in water-treated induced *Aldh1b1^−/−^* Msh2-LS mice (*P*<0.0001, unpaired two-tailed Student's *t*-test) (Fig. 3B), and in ethanol-treated compared with that in water-treated induced *Aldh1b1^flox/flox^* Msh2-LS mice (*P*=0.0006, unpaired two-tailed Student's *t*-test) (Fig. 3A). The percentage of MSH2-negative crypts was also significantly higher in the SI of both ethanol-treated induced *Aldh1b1^flox/flox^* Msh2-LS and *Aldh1b1^−/−^* Msh2-LS mice compared with that in the relevant water-treated mice, and there were more MSH2-negative crypts in the entire SI than in the entire colon (Fig. S6A-G). However, no tumours were observed in the SI in any mice. Significantly higher numbers of colonic MSH2-negative crypts were found in both ethanol-treated induced *Aldh1b1^−/−^* Msh2-LS and *Aldh1b1^flox/flox^* Msh2-LS mice compared with those in similarly treated *Aldh1b1^wt^* Msh2-LS mice (previous data, Cerretelli et al., 2021) (*P*=0.0262 and *P*<0.0001, respectively, unpaired two-tailed Student's *t*-test), with more MSH2-negative crypts in ethanol-treated induced *Aldh1b1^−/−^* Msh2-LS mice than in *Aldh1b1^flox/flox^* Msh2-LS mice (*P*=0.004, unpaired two-tailed Student's *t*-test) (Fig. 3M). No MSH2-negative crypts were observed in the intestinal samples of both non-induced *Aldh1b1^flox/flox^* Msh2-LS and non-induced *Aldh1b1^−/−^* Msh2-LS control mice (either ethanol- or water-treated) (Fig. S7A-H).

ALDH1B1-negative crypts were observed scattered along the entire SI and LI of ethanol-treated (and water-treated) induced *Aldh1b1^flox/flox^* Msh2-LS mice (Fig. 1F; Fig. S8A, Fig. S9A,C). By contrast, no ALDH1B1-negative crypts were observed in the intestinal tissues from non-induced *Aldh1b1^flox/flox^* Msh2-LS mice (ethanol- or water-treated) (Fig. 1F; Fig. S8B, Fig. S9B,D). Immunohistochemistry (IHC) analysis of ALDH1B1 expression in the intestinal tissues from both ethanol-treated (or water-treated) induced and non-induced *Aldh1b1^−/−^* Msh2-LS mice showed only ALDH1B1-negative crypts (Fig. 1F; Fig. S8C,D, Fig. S9E-H). ALDH1B1 immunostaining showed that the colonic adenomas tested from ethanol-treated induced *Aldh1b1^flox/flox^* Msh2-LS mice displayed ALDH1B1-negative dysplastic glands.

### Ethanol/acetaldehyde induces colonic epithelial proliferation and β-catenin expression changes

The proportion of intestinal epithelial cells positive for Ki-67 (a proliferation marker) was determined in relation to the total crypt length (Fig. 2C,D, Fig. 3C-F; Fig. S5C,D) (Bankhead et al., 2017). The Ki-67^+^ cell proportion per crypt was significantly higher in colons from ethanol-treated induced *Aldh1b1^−/−^* Msh2-LS mice compared with that in water-treated induced *Aldh1b1^−/−^* Msh2-LS mice (*P*<0.0001, unpaired two-tailed Student's *t*-test) (Fig. 3D), and a similar difference was observed in ethanol-treated versus water-treated induced *Aldh1b1^flox/flox^* Msh2-LS mice (*P*<0.0001, unpaired two-tailed Student's *t*-test) (Fig. 3C). Similar findings were observed in the SI (Fig. S10A-F).

Expression of the β-catenin protein (CTNNB1) was investigated by IHC in colonic tumours from ethanol-treated induced *Aldh1b1^−/−^* Msh2-LS mice, which showed a heterogeneous pattern with variable numbers of adenoma cells with moderately to strongly positive β-catenin nuclear immunostaining owing to accumulation and translocation of β-catenin into tumour nuclei (Fig. 2E,F). This was also observed in ethanol-treated induced *Aldh1b1^flox/flox^* Msh2-LS murine colonic adenomas (Fig. S5E,F), indicating activation of the Wnt signalling pathway in these adenomas, compared with very low levels of nuclear β-catenin expression in the normal mucosal epithelium (Fig. S11).

### Acetaldehyde is associated with colonic DNA damage response and epithelial apoptosis

To investigate the potential for acetaldehyde-induced DNA alterations, IHC was performed for the DNA damage response biomarkers γ-H2AX and p53 (encoded by *Tp53*) (Fig. 2G-J; Fig. S5G-J). γ-H2AX immunostaining showed high expression levels in the ethanol-treated induced *Aldh1b1^−/−^* Msh2-LS large intestinal adenomas (Fig. 2N). The percentage of γ-H2AX-positive cells was significantly higher in ethanol-treated induced *Aldh1b1^−/−^* Msh2-LS non-tumour-bearing colonic mucosal epithelia (46%) compared with that in water-treated induced *Aldh1b1^−/−^* Msh2-LS colonic epithelia (0.5%) (*P*<0.0001, unpaired two-tailed Student's *t*-test) (Fig. 2G,H, Fig. 3G,H). The percentage of SI γ-H2AX-positive cells was higher in the ethanol-treated induced *Aldh1b1^−/−^* Msh2-LS mice (3.4%) compared with that in water-treated induced *Aldh1b1^−/−^* Msh2-LS mice (0.1%) (Fig. S12C-E). Similar findings were observed in ethanol-treated versus water-treated colonic mucosal epithelia of induced *Aldh1b1^flox/flox^* Msh2-LS (*P*<0.0001, unpaired two-tailed Student's *t*-test), but no γ-H2AX-positive cells were observed in the SI of these mice (Fig. 3G; Fig. S5G,H,N, Fig. S12A,B).

Ethanol-treated induced *Aldh1b1^−/−^* Msh2-LS colonic adenomas showed widespread variably high p53 expression (Fig. 2O) reflecting the ‘wild-type pattern’ in response to ethanol-induced genotoxic damage (Köbel et al., 2016; Lakin and Jackson, 1999). No tumours showed either the ‘overexpression’ or ‘null’ patterns associated with *Tp53* mutation. A significantly higher proportion of p53-positive cells with high to moderate nuclear staining was observed in ethanol-treated induced *Aldh1b1^−/−^* Msh2-LS (non-tumour bearing) colonic mucosal epithelia (47.5%) compared with that in water-treated induced *Aldh1b1^−/−^* Msh2-LS colonic epithelia (3.3%) (*P*<0.0001, unpaired two-tailed Student's *t*-test) (Fig. 2I,J, Fig. 3J). Similar findings were observed in ethanol-treated versus water-treated induced *Aldh1b1^flox/flox^* Msh2-LS mice (Fig. 3I; Fig S5I,J,O). The percentage of p53-positive cells in the SI was higher in ethanol-treated induced *Aldh1b1^flox/flox^* and *Aldh1b1^−/−^* Msh2-LS mice compared with that in water-treated induced *Aldh1b1^flox/flox^* and *Aldh1b1^−/−^* Msh2-LS mice, respectively (Fig. S13A-F).

To detect apoptotic events, IHC analysis of cleaved caspase-3 (cCas3) was performed (Fig. 2K,L; Fig S5K,L) (Bankhead et al., 2017; Toft et al., 1999). Ethanol-treated induced *Aldh1b1^−/−^* Msh2-LS murine colonic adenomas showed no or almost no detectable cCas3^+^ apoptotic bodies, indicating rare to no apoptotic events in defective mismatch repair (dMMR) colonic tumours (Fig. 2P). IHC of cCas3 showed significantly higher numbers of cCas3^+^ apoptotic bodies in the ethanol-treated induced *Aldh1b1^−/−^* Msh2-LS non-tumour-bearing colon compared with no or almost no detectable cCas3^+^ apoptotic bodies in the water-treated induced *Aldh1b1^−/−^* Msh2-LS colon (*P*=0.0026, unpaired two-tailed Student's *t*-test) (Fig. 2K,L, Fig. 3L). Similar findings were observed in ethanol-treated versus water-treated induced *Aldh1b1^flox/flox^* Msh2-LS mice (Fig. 3K; Fig. S5K,L,P).

**Fig. 4.**
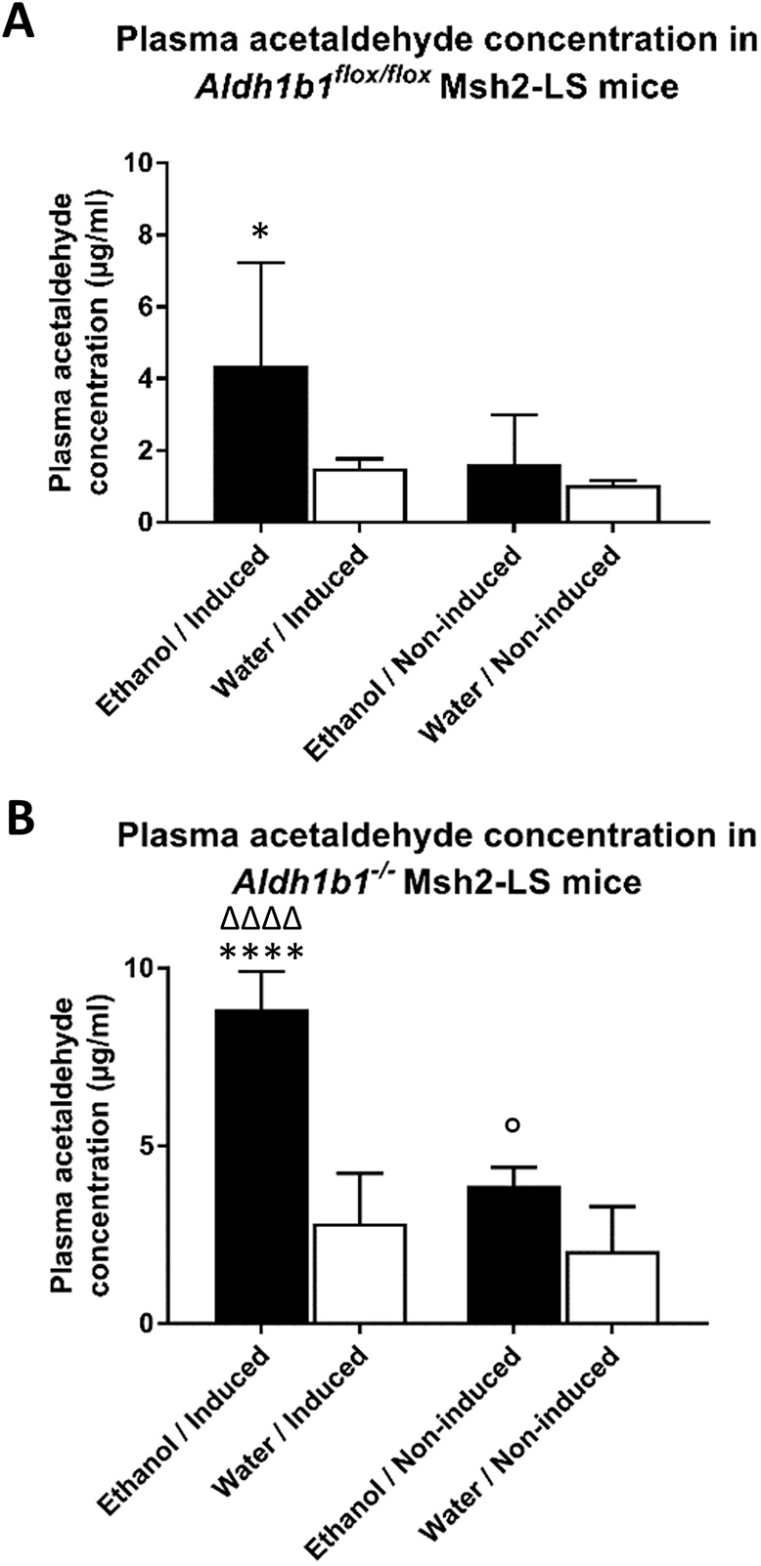
**Plasma acetaldehyde concentrations in ethanol-treated and water-treated *Aldh1b1^flox/flox^* Msh2-LS and *Aldh1b1^−/−^* Msh2-LS mice.** (A) Plasma acetaldehyde concentrations in induced and non-induced *Aldh1b1^flox/flox^* Msh2-LS mice; Mann–Whitney *U*-test, *P=0.0159 for ethanol-treated induced *Aldh1b1^flox/flox^* Msh2-LS mice versus water-treated induced *Aldh1b1^flox/flox^* Msh2-LS mice. (B) Plasma acetaldehyde concentrations in induced and non-induced *Aldh1b1^−/−^* Msh2-LS mice; Mann–Whitney *U*-test, ****P<0.0001 for ethanol-treated induced *Aldh1b1^−/−^* Msh2-LS mice versus water-treated induced *Aldh1b1^−/−^* Msh2-LS mice; ^ΔΔΔΔ^*P*<0.0001 for ethanol-treated induced *Aldh1b1^−/−^* Msh2-LS mice versus ethanol-treated non-induced *Aldh1b1^−/−^* Msh2-LS mice; °*P*=0.0362 for ethanol-treated non-induced *Aldh1b1^−/−^* Msh2-LS mice versus water-treated non-induced *Aldh1b1^−/−^* Msh2-LS mice. Data are shown as mean±s.d., *n*=4-6.

The increased DNA damage response observed in the murine colonic mucosal epithelium is consistent with higher levels of circulating acetaldehyde that were detected by the plasma acetaldehyde assay. Plasma acetaldehyde levels were statistically significantly higher in ethanol-treated induced *Aldh1b1^−/−^* Msh2-LS mice compared with those in water-treated induced *Aldh1b1^−/−^* Msh2-LS mice (*P*<0.0001, Mann–Whitney *U*-test); a similar difference was observed in non-induced *Aldh1b1^−/−^* Msh2-LS mice (*P*=0.0362, Mann–Whitney *U*-test) (Fig. 4B). Plasma acetaldehyde levels in ethanol-treated induced *Aldh1b1^−/−^* Msh2-LS mice were significantly higher compared with those in ethanol-treated non-induced *Aldh1b1^−/−^* Msh2-LS mice (*P*<0.0001, Mann–Whitney *U*-test). In *Aldh1b1^flox/flox^* Msh2-LS mice, statistically significant differences were observed only between ethanol-treated induced *Aldh1b1^flox/flox^* Msh2-LS mice and water-treated induced *Aldh1b1^flox/flox^* Msh2-LS mice (*P*=0.0159, Mann–Whitney *U*-test) (Fig. 4A).

## DISCUSSION

In this study, the conditional knockout *Aldh1b1^flox/flox^* alleles and constitutive knockout *Aldh1b1^−/−^* alleles were introduced by cross-breeding into the Msh2-LS mouse model of LS, in order to study the interactive effects of dMMR with increased acetaldehyde on intestinal tumour formation in LS model mice placed on long-term ethanol treatment. Conditional and constitutive knockout *Aldh1b1* mice had moderate and high plasma levels, respectively, of acetaldehyde relative to water-treated controls and wild-type mice (Müller et al., 2016), as a surrogate indicator of increased concentrations of intestinal intraepithelial acetaldehyde, a highly reactive small aldehyde previously shown to mediate DNA damage (Müller et al., 2016; Cerretelli et al., 2021; Garaycoechea et al., 2012; Langevin et al., 2011).

Ethanol-treated conditional *Aldh1b1^flox/flox^* Msh2-LS mice showed colonic epithelial hyperproliferation and colonic tumours in 42% of animals that were treated with ethanol in an average of 4.5 months, statistically significantly greater than 0% of control water-treated conditional *Aldh1b1^flox/flox^* Msh2-LS mice showing colonic hyperproliferation/tumour formation. An even greater and statistically significant difference was observed for ethanol-treated constitutive *Aldh1b1^−/−^* Msh2-LS mice showing colonic hyperproliferation and tumour formation (67% of animals), in an average of 6 months of ethanol treatment, compared with 0% control water-treated constitutive *Aldh1b1^−/−^* Msh2-LS mice with hyperproliferation/neoplasm formation. No SI tumours were seen in any of the mice. None of the non-induced mice showed SI or colonic tumour formation. The numbers of tumours per tumour-bearing mouse were statistically significantly greater in both ethanol-treated induced conditional *Aldh1b1^flox/flox^* Msh2-LS and ethanol-treated induced constitutive *Aldh1b1^−/−^* Msh2-LS mice compared with those in ethanol-treated induced *Aldh1b1^wt^* Msh2-LS mice. Given that significantly higher plasma levels of acetaldehyde were present in both ethanol-treated (compared with water-treated) induced conditional and constitutive *Aldh1b1* Msh2-LS mice, these experiments provide strong evidence for a gene/environment interaction between dMMR and acetaldehyde in accelerating colonic neoplasm formation, and for a relationship between higher numbers of colonic neoplasms and increasing acetaldehyde levels, indicating a dose-dependent acetaldehyde-tumour association.

Investigation of precursor dMMR lesions in the Msh2-LS murine intestines showed that induced Cre activation caused loss of MSH2 expression in LGR5-expressing crypt epithelial stem cells, subsequently involving whole crypts, scattered along the entire SI and LI. Ethanol-treated induced conditional *Aldh1b1^flox/flox^* Msh2-LS mice showed 43% MSH2-negative SI crypts and 17% MSH2-negative colonic crypts that were statistically significantly greater than the 24% (SI) and 7% (LI) dMMR crypts in water-treated induced *Aldh1b1^flox/flox^* Msh2-LS control mice. Similarly, ethanol-treated induced constitutive *Aldh1b1^−/−^* Msh2-LS mice showed 55.7% MSH2-negative SI crypts and 29% MSH2-negative colonic crypts, statistically significantly greater than the 28% (SI) and 10% (LI) dMMR crypts in water-treated induced *Aldh1b1^−/−^* Msh2-LS mice. There were more MSH2-negative colonic crypts in ethanol-treated induced constitutive *Aldh1b1^−/−^* Msh2-LS mice than in comparable conditional *Aldh1b1^flox/flox^* Msh2-LS mice, and both had more compared with those in similarly treated *Aldh1b1^wt^* Msh2-LS mice, demonstrating a dose-dependent relationship between increasing colonic dMMR precursors and increasing acetaldehyde levels. These data suggest that acetaldehyde causes preferential survival and expansion of dMMR crypt precursors in these LS models. Although the numbers of dMMR crypts were significantly higher in the SI than in the colon in both models, no SI tumours formed in either model, consistent with additional protective mechanisms operating in the SI.

All colonic adenomas tested from ethanol-treated induced conditional *Aldh1b1^flox/flox^* Msh2-LS mice and constitutive *Aldh1b1^−/−^* Msh2-LS mice showed MSH2-negative dysplastic glands, surrounded by MSH2-positive non-neoplastic crypts. This confirmed that colonic adenomas arose from dMMR (MSH2-negative) crypt precursors. This is consistent with observations from human LS patients that the risk of colonic tumour formation correlates with the size of the MMR-deficient crypt clusters that grow over time in affected patients (Wojciechowicz et al., 2014; Cerretelli et al., 2021; Kloor et al., 2012; Shia et al., 2015). Expression patterns of the β-catenin protein showed variably moderately to strongly positive nuclear β-catenin localisation, indicating Wnt pathway activation in adenomas in these models, similar to that observed in human LS tumours (Cong et al., 2003; Arnold et al., 2020).

In both induced *Aldh1b1^flox/flox^* Msh2-LS and *Aldh1b1^−/−^* Msh2-LS mice, the percentage of Ki-67-positive cells per crypt was significantly higher in the colons of ethanol-treated mice compared with that in water-treated mice, confirming increased proliferation in large regions of colonic mucosal crypt elongation identified morphologically as hyperproliferative zones. Such hyperproliferative zones were only seen in ethanol-treated induced (and non-induced) murine colonic epithelial mucosa; they were not observed in the SI of these mice, nor in the LI or SI of water-treated induced or non-induced Msh2-LS mice. This is consistent with ethanol-induced colonic crypt epithelial hyperproliferation that has previously been described by our group (and others) after long-term ethanol-treatment of wild-type mice and ALDH1B1-depleted mice (Müller et al., 2016; Cerretelli et al., 2021; Balbo and Brooks, 2015; Brooks and Zakhari, 2014).

Ethanol metabolism to acetaldehyde plays a major role in intestinal carcinogenesis (Seitz and Stickel, 2007; Brooks, 1997). Acetaldehyde is the first product of ethanol metabolism, and aldehydes are very reactive small molecules that can cause a wide range of DNA modifications (Balbo and Brooks, 2015; Brooks and Zakhari, 2014; Seitz and Stickel, 2007; Brooks, 1997). In normal tissues, acetaldehyde is oxidised to acetate by aldehyde dehydrogenases. ALDH1B1 is the major aldehyde dehydrogenase in the intestinal epithelium (Stagos et al., 2010). Induced *Aldh1b1^flox/flox^* Msh2-LS mice had scattered intestinal crypts lacking ALDH1B1 expression and moderate plasma acetaldehyde levels, whereas *Aldh1b1^−/−^* Msh2-LS mice lacked ALDH1B1 expression in all cells and had markedly increased plasma levels of acetaldehyde, likely leading to accumulation of acetaldehyde-induced DNA damage. The DNA damage response was evaluated by IHC for γ-H2AX and p53, which both showed significantly increased levels in ethanol-treated induced *Aldh1b1^flox/flox^* Msh2-LS and *Aldh1b1^−/−^* Msh2-LS murine colonic mucosal epithelia compared with those in the water-treated equivalent control epithelia, but there were fewer differences in the SI. This increased colonic γ-H2AX and p53 expression is consistent with DNA damage brought about by acetaldehyde following ethanol exposure, with a significant mutagenic effect mainly on dMMR colonic mucosal epithelia, as seen in the adenomas. This suggests that MSH2 and ALDH1B1 play key roles in protecting MMR-proficient colonic epithelia against this type of acetaldehyde-induced DNA damage, but there are additional mechanisms protecting small intestinal epithelial cells from acetaldehyde-induced DNA damage.

MMR is involved in a signalling cascade that leads to either cell cycle arrest or apoptosis if severe DNA damage has occurred. MMR-deficient cells show predisposition to malignancy by failing to repair DNA damage (recognised by the MMR pathway) and are unable to engage apoptosis to eliminate such DNA-damaged cells (Toft et al., 1999; Levine, 1997; Williams and Schumacher, 2016; Cerretelli et al., 2020). Whereas ethanol exposure induced significantly increased apoptosis of predominantly MMR-proficient normal-appearing colonic epithelia in ethanol-treated compared with water-treated induced *Aldh1b1^flox/flox^* Msh2-LS and *Aldh1b1^−/−^* Msh2-LS mice, almost no cCas3^+^ apoptotic bodies were observed in ethanol-treated induced *Aldh1b1^flox/flox^* Msh2-LS and *Aldh1b1^−/−^* Msh2-LS murine dMMR colonic adenomas, indicating almost complete failure of acetaldehyde-mediated DNA damage to induce apoptosis in dMMR colonic tumours. This is consistent with our previous observations in *Msh2*-null murine intestinal epithelia treated with temozolomide, an agent that causes DNA damage recognised by the MMR pathway, that demonstrated the requirement for a functional MMR pathway for apoptosis induction by temozolomide (Toft et al., 1999).

**Fig. 5.**
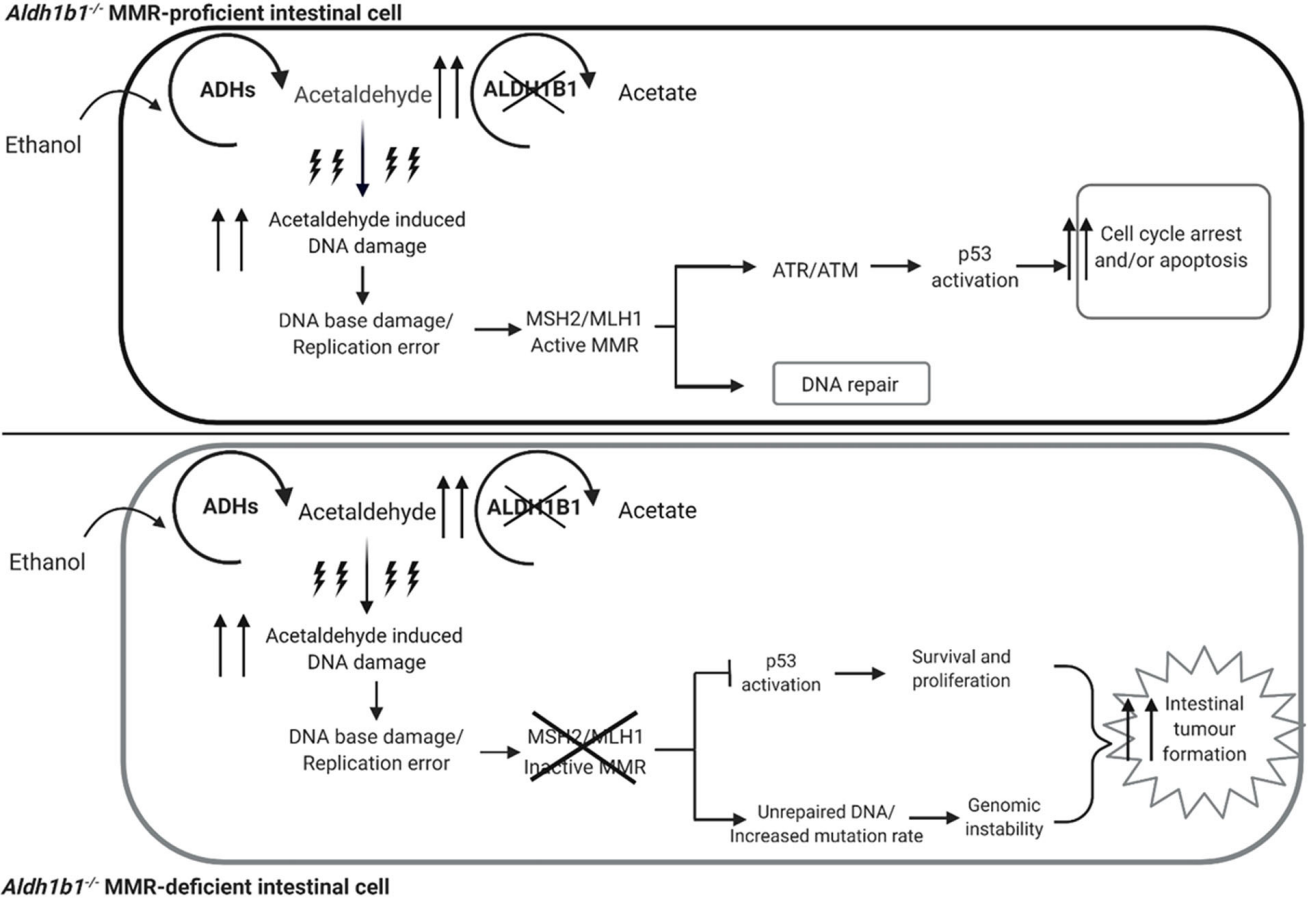
**Schematic of the explanatory model of the MMR/acetaldehyde gene/environment interactions in *Aldh1b1^−/−^* MMR-proficient and *Aldh1b1^−/−^* MMR-deficient colonic epithelial cells.** Ethanol is metabolised to highly reactive acetaldehyde by alcohol dehydrogenases (ADHs) in the colonic epithelium. Acetaldehyde is further oxidised to acetate by aldehyde dehydrogenases, primarily by ALDH1B1 in epithelial stem cells and transit-amplifying cells. In some epithelial cells, acetaldehyde can escape the metabolic pathway and induce various forms of DNA damage, some of which would normally be recognised and repaired by the MMR system, or, if unrepaired, this damage may induce replication errors, such as base mismatches or indels, during the S phase of the cell cycle. In the *Aldh1b1^−/−^* MMR-proficient colonic epithelial cell (upper panel), ethanol is metabolised to highly reactive acetaldehyde, with reduced oxidation to acetate owing to the lack of ALDH1B1 activity, leading to increased levels of highly reactive acetaldehyde that can damage DNA. The *Aldh1b1^−/−^* MMR-proficient colonic epithelial cell is able to activate DNA MMR, inducing either cell cycle arrest (to allow DNA repair) or apoptosis. By contrast, the *Aldh1b1^−/−^* MMR-deficient colonic epithelial cell (lower panel) is unable to activate the MMR signalling pathway, and thus there is neither cell cycle arrest nor apoptosis, following increased acetaldehyde-induced DNA damage, resulting in aberrant survival of DNA-damaged cells and their subsequent proliferation. These proliferating *Aldh1b1^−/−^* MMR-deficient epithelial cells populate the crypt and expand to form *Aldh1b1^−/−^* dMMR crypt foci, the precursor lesions of neoplasms. Stimulated by ethanol/acetaldehyde to undergo increased proliferation, these cells form hyperproliferative crypts whilst remaining subject to ongoing DNA damage. Thus, these *Aldh1b1^−/−^* dMMR cells can accumulate mutations reflecting a form of dMMR genomic instability, and are consequently at increased risk of tumour formation, thus explaining the acceleration of colonic adenoma formation and increased probability of evolution to adenocarcinoma. In the *Aldh1b1^flox/flox^* Msh2-LS model mice, scattered *Aldh1b1^flox/flox^ Msh2^flox/−^* colonic epithelial stem cells are induced by tamoxifen-mediated Cre activation to become A*ldh1b1^−/−^ Msh2^−/−^* MMR-deficient colonic epithelial cells, and thus respond to ethanol exposure in a similar way to that described above.

In conclusion, ethanol treatment was shown to induce zones of hyperproliferation of the colonic, but not the small intestinal, mucosal epithelium, and this appears to contribute to intestinal adenoma formation by acting as a tumour promoter. Tumours occurred mostly in the parts of the colon (proximal and mid-colon) affected by hyperproliferation in these conditional *Aldh1b1^flox/flox^* Msh2-LS and constitutive *Aldh1b1^−/−^* Msh2-LS mice. In both the *Aldh1b1^flox/flox^* Msh2-LS and *Aldh1b1^−/−^* Msh2-LS mice, acetaldehyde-mediated DNA damage and carcinogenic effects appeared to be stronger than those previously observed in the *Aldh1b1^wt^* Msh2-LS mouse model (Cerretelli et al., 2021). Acetaldehyde was shown to cause colonic mucosal epithelial DNA damage responses, but less so in the SI, as observed by IHC for both γ-H2AX and p53. This study produced strong evidence to support the hypothesis that there is a gene/environment interaction between dMMR and acetaldehyde, demonstrated most notably by the *Aldh1b1^−/−^* Msh2-LS mouse model. We propose an explanatory model (Fig. 5) in which the *Aldh1b1^−/−^* MMR-proficient intestinal epithelium metabolises ethanol to highly reactive acetaldehyde, with reduced oxidation to acetate owing to the lack of ALDH1B1, leading to increased levels of highly reactive acetaldehyde that can damage DNA. In *Aldh1b1^−/−^* MMR-proficient colonic epithelial stem cells, acetaldehyde-induced DNA damage is likely to result in cell cycle arrest and DNA repair by the DNA MMR pathway in the case of mild DNA damage, or cell death by apoptosis in the case of severe DNA damage. By contrast, in *Aldh1b1^−/−^* colonic epithelial stem cells with dMMR, significant acetaldehyde-induced DNA damage is not recognised by the DNA MMR system, with no activation of either cell cycle arrest, DNA MMR or apoptosis. The DNA-damaged *Aldh1b1^−/−^* dMMR cells show inappropriate survival and subsequent ethanol-induced proliferation. This leads to an increase of *Aldh1b1^−/−^* dMMR colonic epithelial cells, observed as higher numbers of clusters of *Aldh1b1^−/−^* dMMR crypts and crypt foci, with subsequent ongoing acetaldehyde-mediated DNA damage and dMMR-conferred hypermutation, leading to accelerated colonic tumour evolution. In the *Aldh1b1^flox/flox^* Msh2-LS model mice, scattered *Aldh1b1^flox/flox^ Msh2^flox/−^* colonic epithelial stem cells are induced by tamoxifen-mediated Cre activation to become *Aldh1b1^−/−^ Msh2^−/−^* MMR-deficient colonic epithelial cells and thus respond to ethanol exposure in a similar way to that described for *Aldh1b1^−/−^* dMMR crypt foci. This dMMR/aldehyde gene/environment interaction, enhancing dMMR-driven colonic tumourigenesis, demonstrated in these mouse models is highly likely to apply to human LS patients, indicating that appropriate lifestyle advice concerning reducing alcohol consumption should be considered for LS patients in order to reduce their risk of colorectal tumour formation.

## MATERIALS AND METHODS

### Generation and ethanol treatment of *Aldh1b1^flox/flox^* and *Aldh1b1^−/−^* Msh2-LS mice

The care and use of experimental animals complied with relevant institutional and UK national animal welfare laws and was carried out under a Home Office licence. *Aldh1b1^tm2a(EUCOMM)Wtsi^* mice (sourced from the EUCOMM project; Müller et al., 2016; Skarnes et al., 2011) (background C57BL/6) were crossed with transgenic *Flp-e* mice (provided by Prof. Ian Jackson, University of Edinburgh, Edinburgh, UK) to generate *Aldh1b1* conditional-knockout (*Aldh1b1^flox/flox^*) mice. Embryos from the *Aldh1b1^flox/flox^* mice were treated with TAT-Cre *in vitro* and implanted into surrogate mothers to generate *Aldh1b1* constitutive-knockout (*Aldh1b1^−/−^*) mice. *Aldh1b1^flox/flox^* and *Aldh1b1^−/−^* mice were crossbred with Msh2-LS mice (*Lgr5-CreER*; *Msh2^flox/−^* mice, provided by Hein Te Riele, The Netherlands Cancer Institute, Amsterdam, The Netherlands, on a mixed background C57BL/6 and FVB) (Wojciechowicz et al., 2014), which were further characterised by our group in previous studies (Cerretelli et al., 2021).

Twenty-four (six males and 18 females) *Aldh1b1^flox/flox^* Msh2-LS mice and 24 (16 males and eight females) *Aldh1b1^−/−^* Msh2-LS mice aged 7-9 weeks received intraperitoneal injections of 0.15 mg tamoxifen (Sigma-Aldrich, St Louis, MO, USA/g body weight on day 1 (for 24 h) and 0.1 mg tamoxifen/g body weight on days 2, 3 and 4; from day 5, mice were provided with either 20% ethanol in drinking water or standard drinking water *ad libitum*. Water-treated mice and ethanol-treated mice (12 mice each) were monitored until an ethanol-treated mouse had to be culled for any clinical sign of intestinal tumour formation (Cerretelli et al., 2021) or displayed >20% loss of body weight, following which an age-matched and treatment duration-matched water-treated control mouse was culled at the same timepoint, consistent with ARRIVE guidelines (Kilkenny et al., 2010; Workman et al., 2010). On occasion, ethanol-treated test mice were removed from the study for body weight loss or ulcerative dermatitis with no intestinal tumours being found. No water-treated control mice developed intestinal tumours, so these were never removed from the study before the matched ethanol-treated test mice.

Fourteen (six males and eight females) *Aldh1b1^flox/flox^* Msh2-LS control mice and twelve (eight males and four females) *Aldh1b1^−/−^* Msh2-LS control mice, aged 7-9 weeks, received intraperitoneal injections of 0.15 mg corn oil/g body weight (without tamoxifen, so there was no induction of Cre recombinase activity) on day 1 and 0.1 mg corn-oil/g body weight on days 2, 3 and 4; from day 5, mice were provided with either 20% ethanol in drinking water or standard drinking water. Both water- and ethanol-treated non-induced age-matched mice were sacrificed when they reached the same timepoints as the ethanol-treated induced mice that were culled for a clinical sign indicative of intestinal tumour formation or >20% body weight loss. Treatments using 20% ethanol in drinking water were previously validated (Müller et al., 2016; de Wind et al., 1995; Wojciechowicz et al., 2014; Cerretelli et al., 2021). None of the mice showed abnormal behaviour or reduced body weight initially, indicating good acceptance of the ethanol regime.

### Tissue collection and analysis

At necropsy, water-treated control and ethanol-treated test mice were investigated identically: blood was collected and the SI, LI, caecum, stomach, oesophagus, liver, hepatobiliary tract, pancreas, lungs, spleen, thymus, lymph nodes and any other organ or tissue showing abnormalities were collected and fixed in 10% neutral-buffered formalin for 24 h. The entire SI and LI were examined both macroscopically and microscopically for the presence of tumours, using three Swiss roll sections of the SI and two Swiss roll sections of the LI for full coverage of the intestines from the gastroduodenal junction to the anal canal (Figs S1 and S3). Tissues were processed and paraffin-embedded to blocks, sectioned using a microtome (Leica Microsystems, Milton Keynes, UK.) and stained with Haematoxylin and Eosin. IHC was performed using anti-MSH2 (ab70270, 1:4000, Abcam), anti-ALDH1B1 (15560-1-AP; 1:500, Proteintech), anti-Ki-67 (ab16667, 1:500, Abcam), anti-β-catenin (610154, 1:100, BD Transduction Laboratories, San Jose, CA, USA), anti-cCas3 (9664, 1:50, Cell Signaling Technology), anti-γH2AX (2577, 1:100, Cell Signaling Technology) and anti-p53 (CM5, 1:100, provided by Dr Phil Coates, Masaryk Memorial Cancer Institute, Brno, Czech Republic) antibodies, as previously described (Müller et al., 2016; Cerretelli et al., 2021). IHC stains were quantified using QuPATH v0.2.0 (Bankhead et al., 2017) for Ki-67, γH2AX, p53 and cCas3 in 30 crypt-villus pairs per SI and in 30 crypts per colon for each of four representative mice per genotype and group examined; whereas MSH2 and ALDH1B1 IHC stains were manually counted for negative crypts along the entire SI and the entire colon for each of three mice per genotype and group examined.

### Acetaldehyde assay

Blood was fractionated by centrifugation at 3000 ***g*** for 15 min at 4°C. Plasma was collected and immediately snap-frozen in liquid nitrogen and stored at −80°C. Plasma acetaldehyde concentrations were determined using an acetaldehyde assay kit (Megazyme, K-ACHYD, Co. Wicklow, Ireland), according to the manufacturer's instructions.

### Statistical analysis

Data were analysed using GraphPad Prism v7.0 software (GraphPad, San Diego, CA, USA) and are shown as mean±s.d. Group data were compared using unpaired two-tailed Student's *t*-test, Mann–Whitney *U*-test or two-way ANOVA with Bonferroni correction test. Association between two categories was assessed by two-sided Fisher's exact test. Percentage survival was analysed by log-rank (Mantel–Cox) test. Differences were considered statistically significant if *P*<0.05.

## Supplementary Material

10.1242/dmm.050240_sup1 Supplementary information
