# Supplementary material for: Acetaldehyde and defective mismatch repair increase colonic tumours in a Lynch syndrome model with Aldh1b1 inactivation
Source: Dis Model Mech. 2023 Jul 31;16(8):dmm050240. doi: 10.1242/dmm.050240 (PMC10417510; doi:10.1242/dmm.050240)
Supplement: Supplementary information [file dmm-16-050240-s1.pdf]

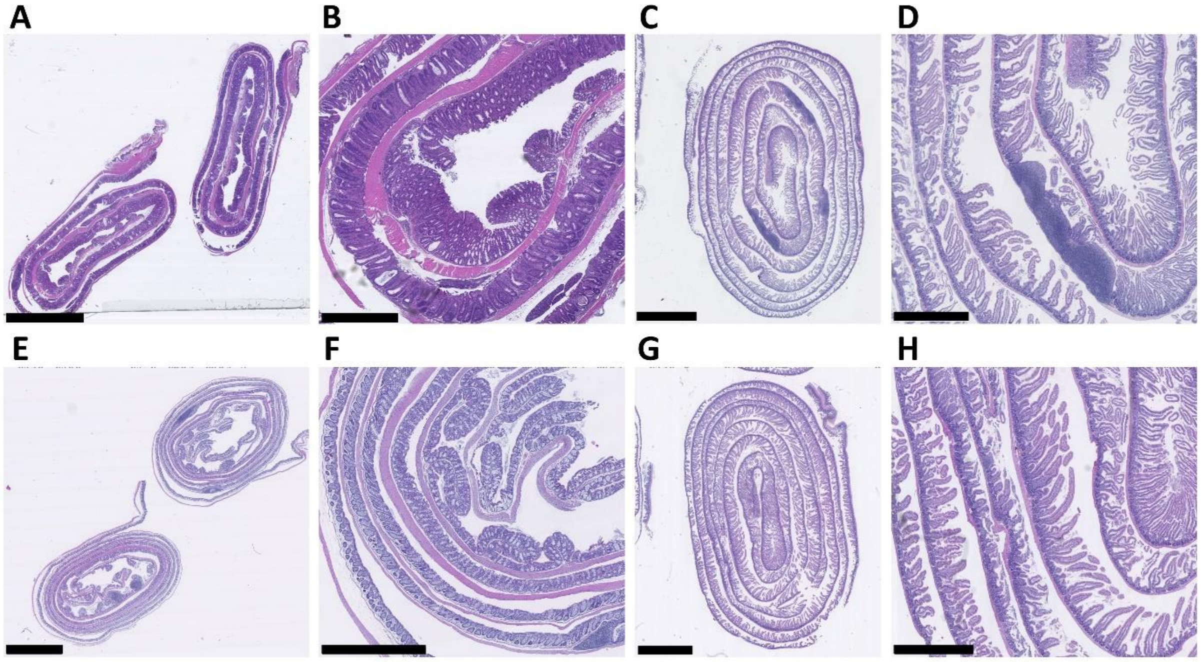

Fig. S1. Representative images of H&E stained colon and SI Swiss rolls. A) Images of H&E stained LI Swiss rolls from ethanol-treated Aldh1b1*flox/flox* Msh2-LS mice, further magnified in image (B) showing widespread hyperproliferation of the elongated colonic crypts. Images taken from scanned slide files with the Hamamatsu Nanozoomer NDP Viewer software at 0.6X and 2.5X magnification respectively (scale bar at lower left indicates 2.5mm in A and 1mm in B). C) Image of H&E stained SI Swiss roll from an ethanol-treated Aldh1b1*flox/flox* Msh2-LS mouse, further magnified in image (D). Images taken from scanned slide files with the Hamamatsu Nanozoomer NDP Viewer software at 0.6X and 2.5X magnification respectively (scale bar at lower left indicates 2.5mm in C and 1mm in D). E) Images of H&E stained LI Swiss rolls from water-treated Aldh1b1*flox/flox* Msh2-LS mice, further magnified in image (F) showing normal large intestinal mucosal appearances (scale bar at lower left indicates 2.5mm in E and 1mm in F) G) Image of H&E stained SI Swiss roll from a water-treated Aldh1b1*flox/flox* Msh2-LS mouse, further magnified in image (H) (scale bar at lower left indicates 2.5mm in G and 1mm in H).

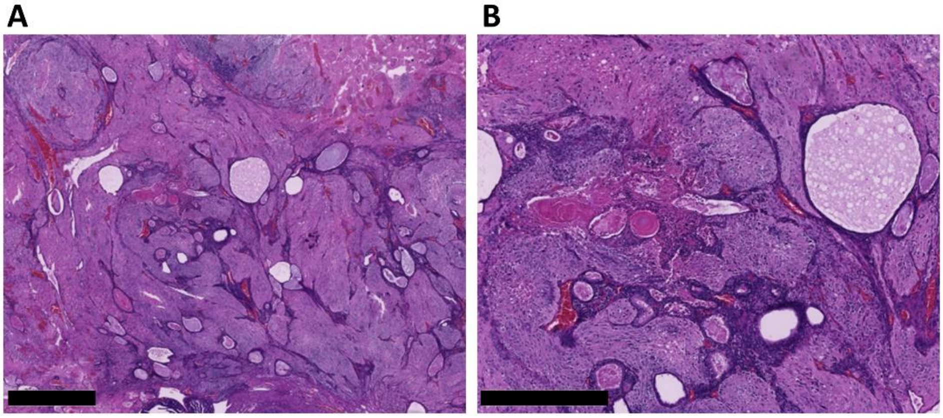

Fig. S2. Representative images of H&E stained uterine endometrial tumour. A) Representative image of uterine endometrial invasive adenocarcinoma from an ethanol-treated induced *Aldh1b1*$^{flox/flox}$ Msh2-LS mouse. Images taken at 22X (A) and 63X (B) magnification (scale bar A: 1mm; scale bar B: 500µm).

Disease Models & Mechanisms • Supplementary information

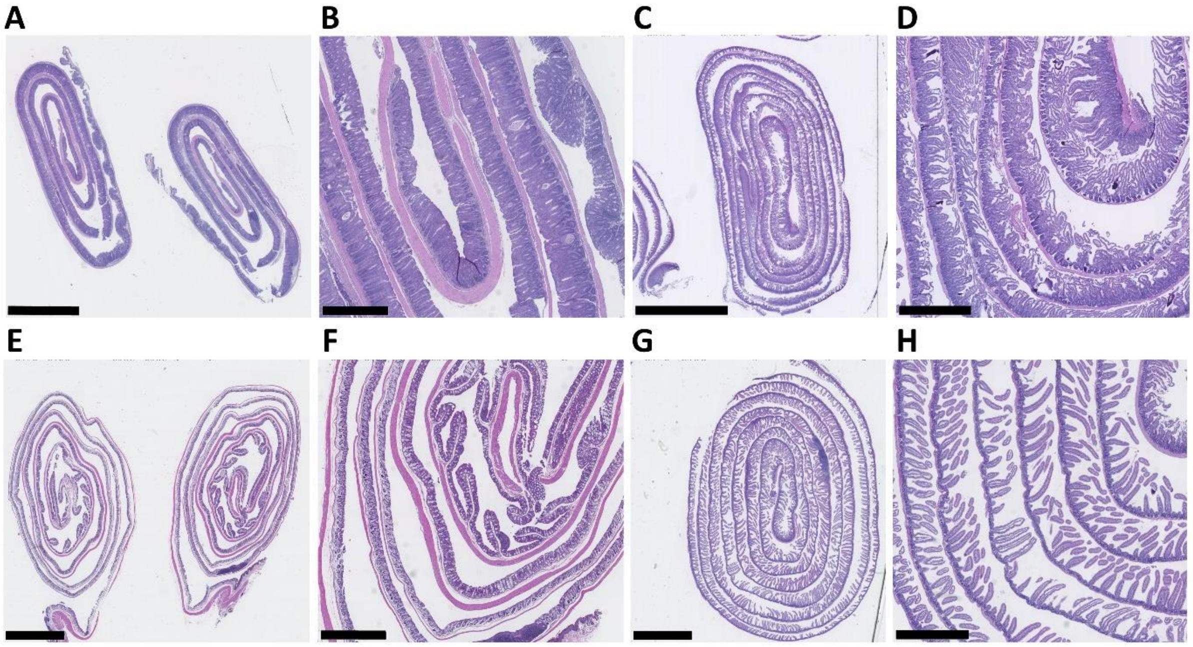

Fig. S3. Representative images of H&E stained colon and SI Swiss rolls. A) Images of H&E stained LI Swiss rolls from ethanol-treated Aldh1b1$^{-/-}$ Msh2-LS mice, further magnified in image (B) showing widespread hyperproliferative changes with colonic crypt elongation. Images taken from scanned slide files with the Hamamatsu Nanozoomer NDP Viewer software at 0.5X and 2.5X magnification respectively (scale bar at lower left indicates 5mm in A and 1mm in B). C) Image of H&E stained SI Swiss roll from an ethanol-treated Aldh1b1$^{-/-}$ Msh2-LS mouse, further magnified in image (D). Images taken from scanned slide files with the Hamamatsu Nanozoomer NDP Viewer software at 0.5X and 2.5X magnification respectively (scale bar at lower left indicates 5mm in C and 1mm in D). All images show normal small intestinal mucosal appearances. E) Images of H&E stained LI Swiss rolls from water-treated Aldh1b1$^{-/-}$ Msh2-LS mice, further magnified in image (F), showing normal large intestinal mucosal appearances. G) Image of H&E stained SI Swiss roll from a water-treated Aldh1b1$^{-/-}$ Msh2-LS mouse, further magnified in image (H). Images taken from scanned slide files with the Hamamatsu Nanozoomer NDP Viewer software at 0.7X and 2.5X (scale bars at lower left indicates 5mm in E and G; and 1mm in F and H). LI: Large Intestine. SI: Small Intestine.

Disease Models & Mechanisms • Supplementary information

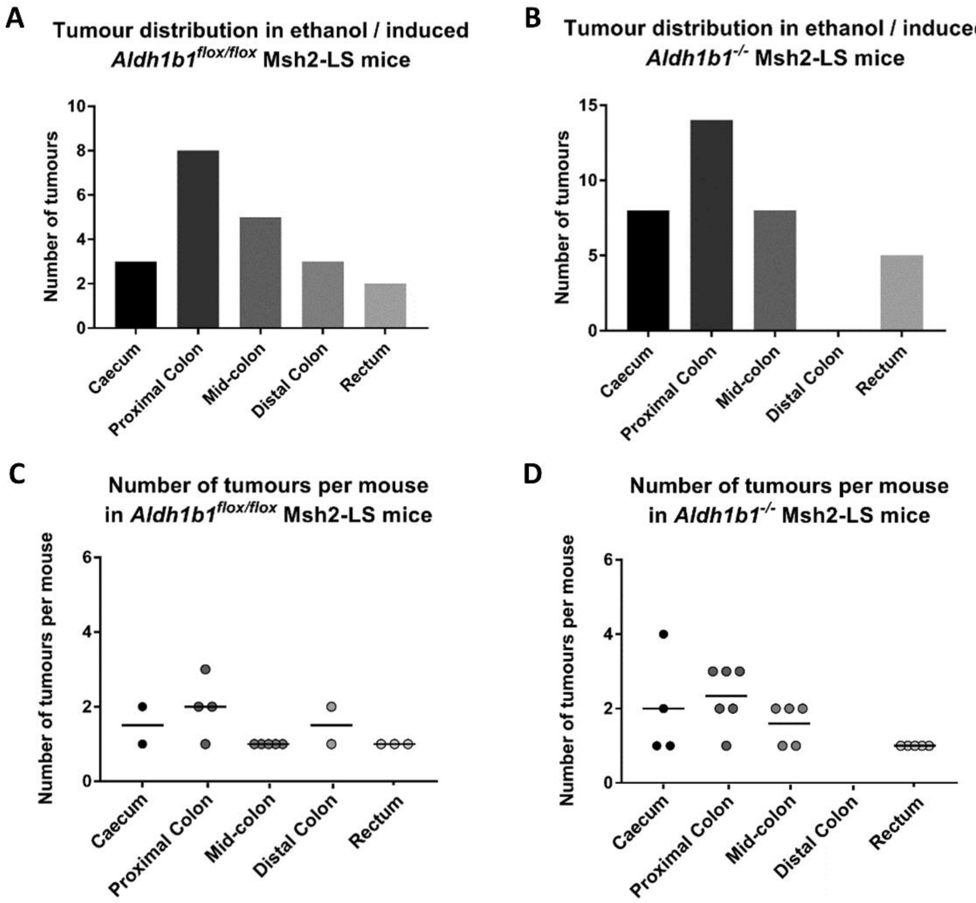

Fig. S4. Tumour distribution and numbers of tumours per mouse for colorectal tumour formation in *Aldh1b1$^{flox/flox}$* Msh2-LS and *Aldh1b1$^{-/-}$* Msh2-LS mice. A) Tumour distribution in ethanol-treated induced *Aldh1b1$^{flox/flox}$* Msh2-LS tumour-bearing mice. In total, 21 neoplasms were observed: 3 adenomas in the caecum; 8 adenomas in the proximal colon (1/8 was an invasive adenocarcinomas); 5 adenomas in the mid-colon; 3 adenomas in the distal colon; and 2 adenomas in the rectum. B) Tumour distribution in ethanol-treated induced *Aldh1b1$^{-/-}$* Msh2-LS tumour-bearing mice. In total, 35 neoplasms were observed: 8 adenomas in the caecum; 14 adenomas in the proximal colon; 8 adenomas in the mid-colon; 0 adenomas in the distal colon; and 5 adenomas in the rectum. C) Tumour distribution and number per mouse in ethanol-treated induced *Aldh1b1$^{flox/flox}$* Msh2-LS tumour-bearing mice: 1/5 tumour-bearing mice showed one caecal adenoma and 1/5 mice showed two adenomas in the caecum; 1/5 tumour-bearing mice showed one proximal colonic adenoma; 2/5 showed two adenomas and 1/5 showed three adenomas in the proximal colon; 5/5 tumour-bearing mice showed one mid-colonic adenoma; 1/5 tumour-bearing mice showed 2 distal colonic adenomas and 1/5 showed one adenoma in the distal colon; 2/5 tumour-bearing mice showed one rectal adenoma. D) Tumour distribution and number per mouse in ethanol-treated induced *Aldh1b1$^{-/-}$* Msh2-LS tumour-bearing mice: 2/8 tumour-bearing mice showed one caecal adenoma, 1/8 mice showed two caecal adenomas and 1/8 showed 4 adenomas in the caecum; 1/8 tumour-bearing mice

Disease Models & Mechanisms • Supplementary information

showed one proximal colonic adenoma, 2/8 showed two adenomas and 3/8 showed three adenomas in the proximal colon; 2/8 tumour-bearing mice showed one mid-colonic adenoma and 3/8 showed 2 adenomas in the mid-colon. No ethanol-treated induced *Aldh1b1*$^{-/-}$ Msh2-LS mice showed adenoma formation in the distal colon. However, 5/8 tumour-bearing mice showed one rectal adenoma. (Horizontal bars represent means).

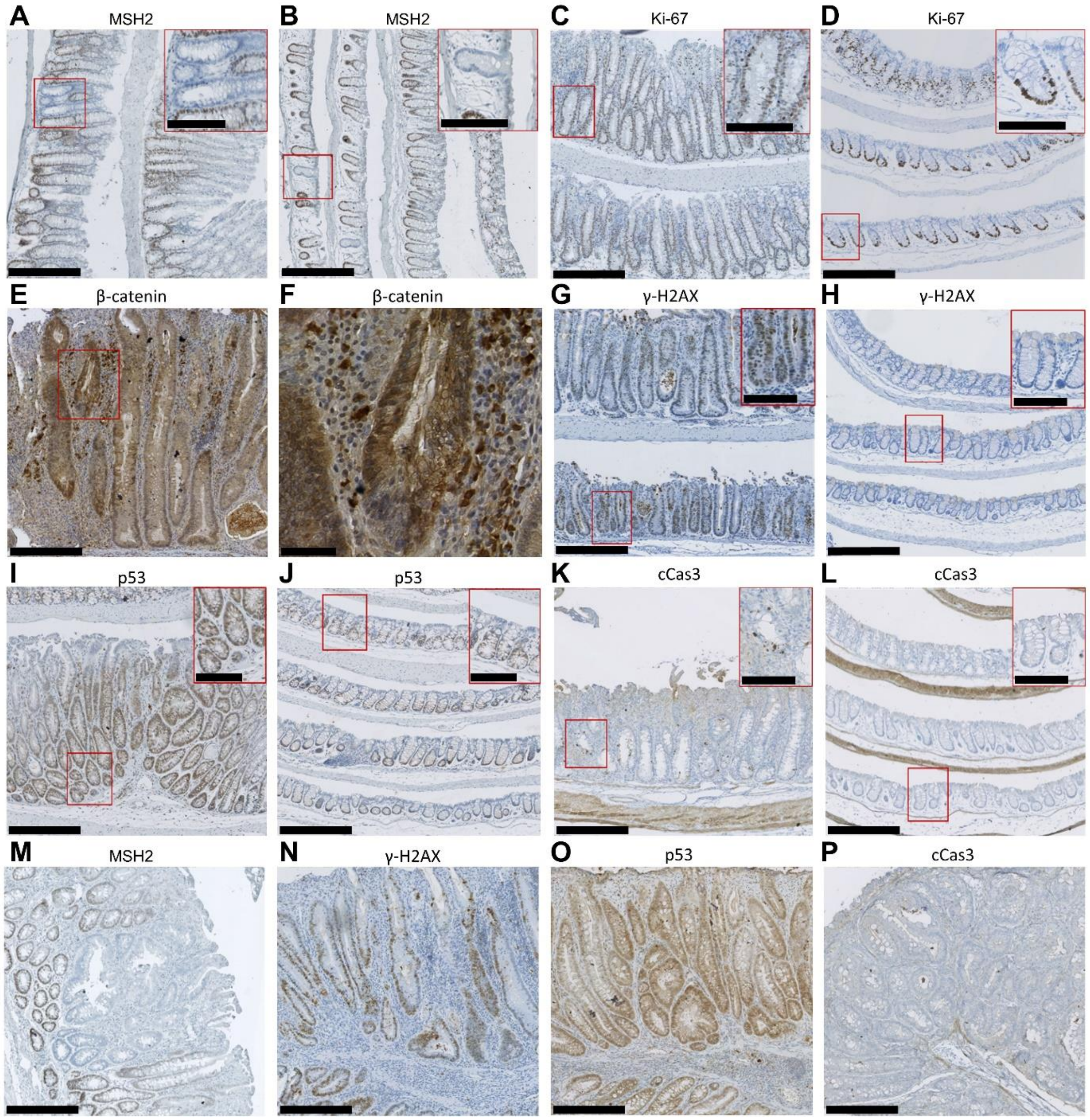

Fig. S5. Representative images of IHC analysis of induced *Aldh1b1*$^{flox/flox}$ Msh2-LS murine colonic mucosal epithelium and adenomas. A, B) MSH2 immunostaining of murine colon showing some MSH2-negative crypts from ethanol-treated induced *Aldh1b1*$^{flox/flox}$ Msh2-LS mice (A) and water-treated induced *Aldh1b1*$^{flox/flox}$ Msh2-LS mice (B). C, D) Ki-67 immunostaining of murine colon from ethanol-treated induced *Aldh1b1*$^{flox/flox}$ Msh2-LS mice showing hyperproliferative crypt elongation (C) and normal colon in water-treated induced *Aldh1b1*$^{flox/flox}$ Msh2-LS mice (D). E, F) Representative image of β-catenin immunostaining in a colonic adenoma from an ethanol-treated induced *Aldh1b1*$^{flox/flox}$ Msh2-LS mouse (E), with selected area (red square) within image E (magnification 100X) further magnified to 400X in image F. G, H) Immunostaining for γ-H2AX in ethanol-treated induced *Aldh1b1*$^{flox/flox}$ Msh2-LS mice (G) and water-treated induced *Aldh1b1*$^{flox/flox}$ Msh2-LS mice (H). I, J) Immunostaining for p53 in ethanol-treated induced *Aldh1b1*$^{flox/flox}$ Msh2-LS mice (I) and water-

Disease Models & Mechanisms • Supplementary information

treated induced *Aldh1b1$^{flox/flox}$* Msh2-LS mice (J). K, L) Immunostaining for cCas3 in ethanol-treated induced *Aldh1b1$^{flox/flox}$* Msh2-LS mice (K) and water-treated induced *Aldh1b1$^{flox/flox}$* Msh2-LS mice (L). All main panels show images taken at magnification of 100X (with further magnification of 200X in the upper right inset red rectangles). M-P) Representative images of colonic adenomas from ethanol-treated induced *Aldh1b1$^{flox/flox}$* Msh2-LS mice immunostained for MSH2 (M), γ-H2AX (N), p53 (O) and cCas3 (P); images taken at 100X magnifications in M, N, O, P. Scale bars A-E, G-P (main panel): 250µm (100µm in inset rectangles), scale bar F: 50µm.

Disease Models & Mechanisms • Supplementary information

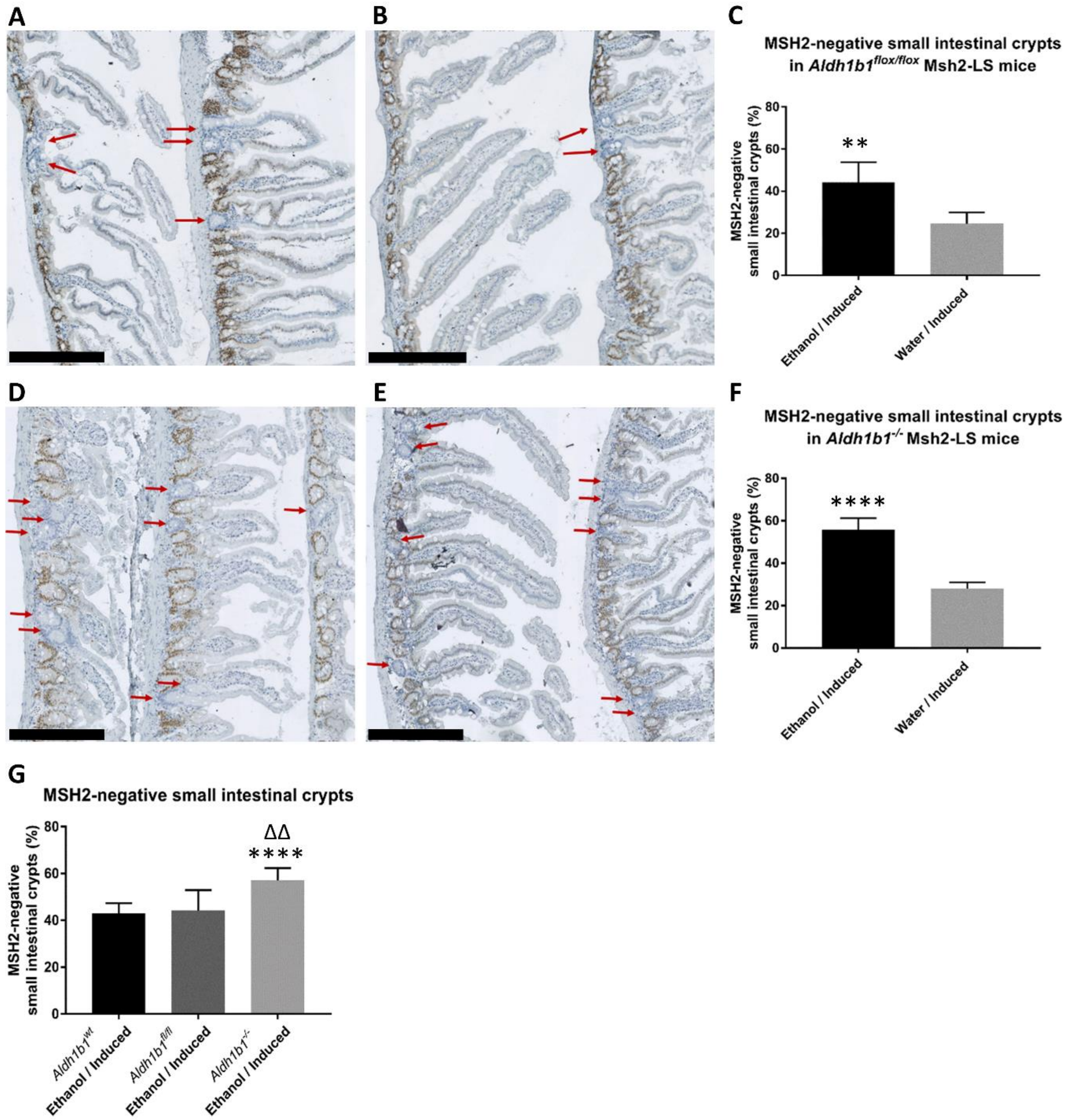

Fig. S6. Representative images of SI immunostained for MSH2 with quantification of numbers of MSH2-negative SI crypts. Immunohistochemical analysis of MSH2 protein expression in small intestinal mucosal epithelium of induced *Aldh1b1*$^{flox/flox}$ Msh2-LS mice treated with either 20% ethanol in drinking water (A) or standard drinking water (B), identifying MSH2-negative crypts (red arrows). C) Percentage of MSH2-negative crypts in small intestinal mucosa of induced *Aldh1b1*$^{flox/flox}$ Msh2-LS mice treated with either 20% ethanol in drinking water or standard water; unpaired two-tailed Students t-test, **p=0.0056 vs. water. Immunohistochemical analysis of MSH2 protein expression in small intestinal mucosal epithelium of induced *Aldh1b1*$^{-/-}$ Msh2-LS mice treated with either 20% ethanol (D) or water (E), identifying MSH2-negative crypts (red arrows). F) Percentage of MSH2-negative crypts in small intestinal mucosa of induced *Aldh1b1*$^{-/-}$ Msh2-LS mice treated with either 20% ethanol or water; unpaired two-tailed Students t-test, ****p<0.0001 vs. water. Data

shown as mean±SD error bars, 300 crypts were analysed in each mouse, n=6 mice for each group. Images taken at 100X magnification (A, B, D and E), scale bars A, B, D, E: 250µm. G) Percentage of MSH2-negative crypts in small intestinal mucosa of ethanol-treated induced *Aldh1b1*$^{wild\text{-}type}$ Msh2-LS mice (previous data from Cerretelli et al, 2021), *Aldh1b1*$^{flox/flox}$ Msh2-LS mice and *Aldh1b1*$^{-/-}$ Msh2-LS mice. Unpaired two-tailed Student's t-test showed statistically significant differences for comparisons of *Aldh1b1*$^{-/-}$ Msh2-LS mice vs. *Aldh1b1*$^{wild\text{-}type}$ Msh2-LS mice, ****p=0.0001 and for *Aldh1b1*$^{-/-}$ Msh2-LS vs. *Aldh1b1*$^{flox/flox}$ Msh2-LS mice, $^{\Delta\Delta}$p=0.0033; but no statistically significant difference was observed between *Aldh1b1*$^{flox/flox}$ Msh2-LS mice and *Aldh1b1*$^{wild\text{-}type}$ Msh2-LS mice. Data shown as mean±SD error bars, 300 crypts were analysed in each mouse, n=6 mice for each group.

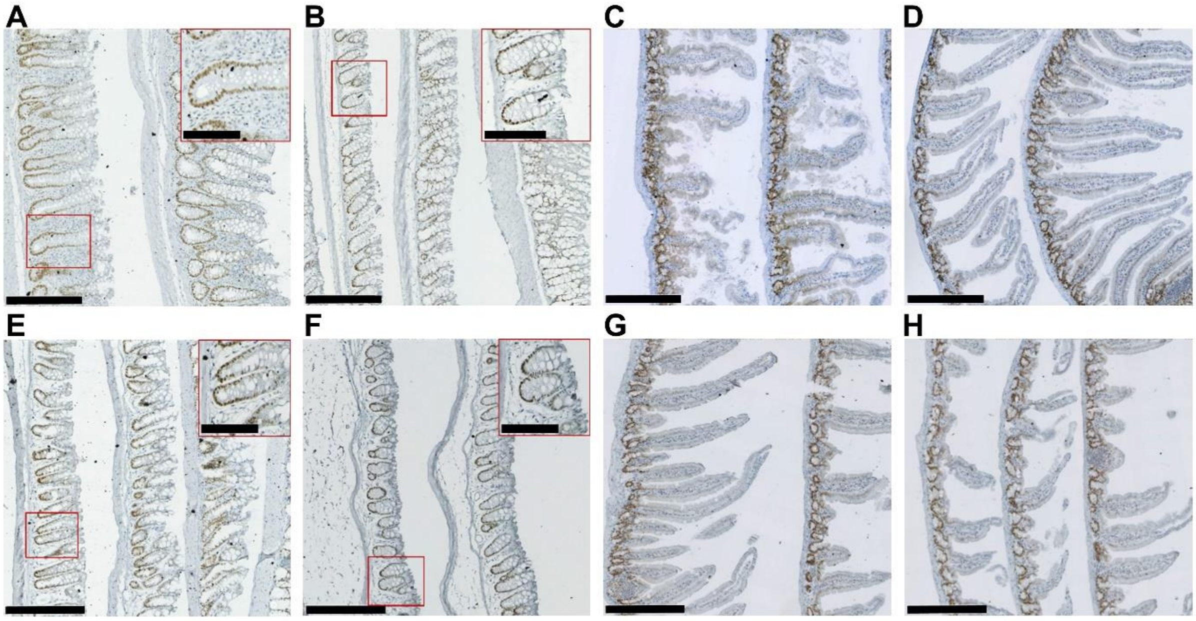

Fig. S7. Representative images of MSH2 immunostained colon and SI Swiss rolls. Immunohistochemical analysis of MSH2 protein expression in large intestinal (A&B) and small intestinal (C&D) mucosal epithelium of non-induced *Aldh1b1*$^{flox/flox}$ Msh2-LS mice treated with either 20% of ethanol (A, C) or water (B, D). Immunohistochemical analysis of MSH2 protein expression in large intestinal (E&F) and small intestinal (G&H) mucosal epithelium of non-induced *Aldh1b1*$^{-/-}$ Msh2-LS mice treated with either 20% of ethanol (E&G) or water (F&H). No MSH2-negative crypts were observed along the entire small and large intestines of either non-induced *Aldh1b1*$^{flox/flox}$ Msh2-LS mice or non-induced *Aldh1b1*$^{-/-}$ Msh2-LS mice (n=6 mice in each group). Magnifications of 100X (main images) and 200X (inset red rectangles). Scale bars A, B, C, D, E, F, G, H: 250µm (inset red rectangles in A, B, E, F: 100µm).

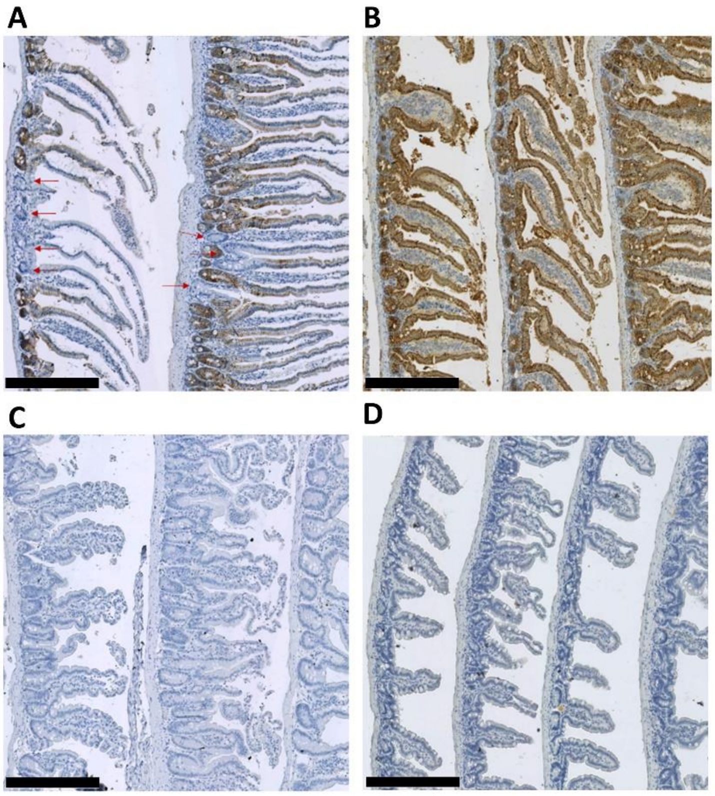

Fig. S8. Representative images of ALDH1B1 immunostained SI. A) Representative image of ALDH1B1 immunostaining of murine small intestinal mucosal epithelium with some ALDH1B1-negative crypts (red arrows) in ethanol-treated induced *Aldh1b1*$^{flox/flox}$ Msh2-LS mice. B) Representative image of ALDH1B1 immunostaining of murine small intestinal mucosal epithelium with all crypts positive for ALDH1B1 in ethanol-treated non-induced *Aldh1b1*$^{flox/flox}$ Msh2-LS mice. C&D) Representative images of ALDH1B1 immunostaining of small intestinal mucosal epithelium with all crypts lacking ALDH1B1 expression in ethanol-treated induced *Aldh1b1*$^{-/-}$ Msh2-LS mice (C) and in ethanol-treated non-induced *Aldh1b1*$^{-/-}$ Msh2-LS mice (D). Images taken at 100X magnification (A, B, C and D), scale bars A, B, C, D: 250µm.

Disease Models & Mechanisms • Supplementary information

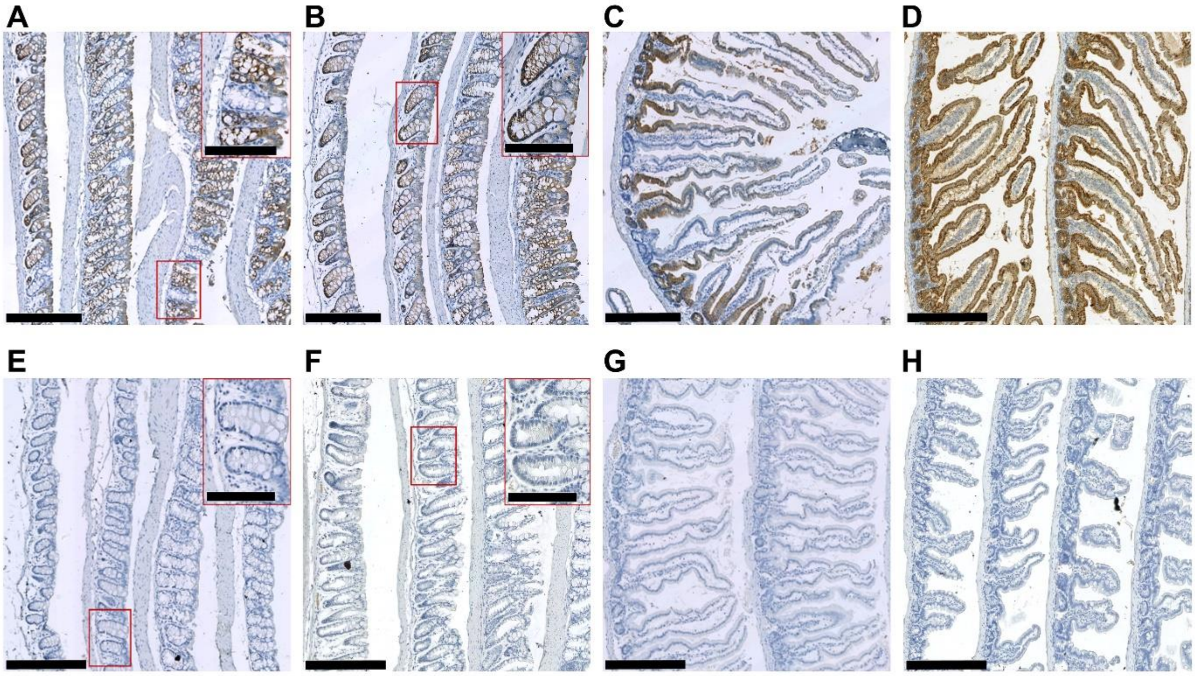

Fig. S9. Representative images of ALDH1B1 immunostained colon and SI. Immunohistochemical analysis of ALDH1B1 protein expression in large intestinal and small intestinal mucosal epithelium of water-treated induced *Aldh1b1*$^{flox/flox}$ Msh2-LS mice (A&C) and water-treated non-induced *Aldh1b1*$^{flox/flox}$ Msh2-LS mice (B&D). Water-treated induced *Aldh1b1*$^{flox/flox}$ Msh2-LS mice showed ALDH1B1-negative crypts in colonic intestinal mucosal epithelium (indicated by the red rectangle and further magnified in the upper right inset red rectangle in figure panel A) and small intestinal mucosal epithelium. No ALDH1B1-negative crypts were observed in either large or small intestinal mucosal epithelium (B and D respectively) of water-treated non-induced *Aldh1b1*$^{flox/flox}$ Msh2-LS mice. Immunohistochemical analysis of ALDH1B1 protein expression in large intestinal and small intestinal mucosal epithelium of water-treated induced *Aldh1b1*$^{-/-}$ Msh2-LS mice (E&G) and water-treated non-induced *Aldh1b1*$^{-/-}$ Msh2-LS mice (F&H). No ALDH1B1 expression was observed in either colonic mucosal epithelium (indicated by the red square and further magnified in the upper right inset red rectangle in figure panels E and F) or small intestinal mucosal epithelium (G&H) of both water-treated induced *Aldh1b1*$^{-/-}$ Msh2-LS mice and water-treated non-induced *Aldh1b1*$^{-/-}$ Msh2-LS mice. Images taken at magnifications of 100X (main images A-H) and 200X (inset red rectangles in A, B, E, F), scale bars A, B, C, D, E, F, G, H: 250µm (inset red rectangles A, B, E, F: 100µm).

Disease Models & Mechanisms • Supplementary information

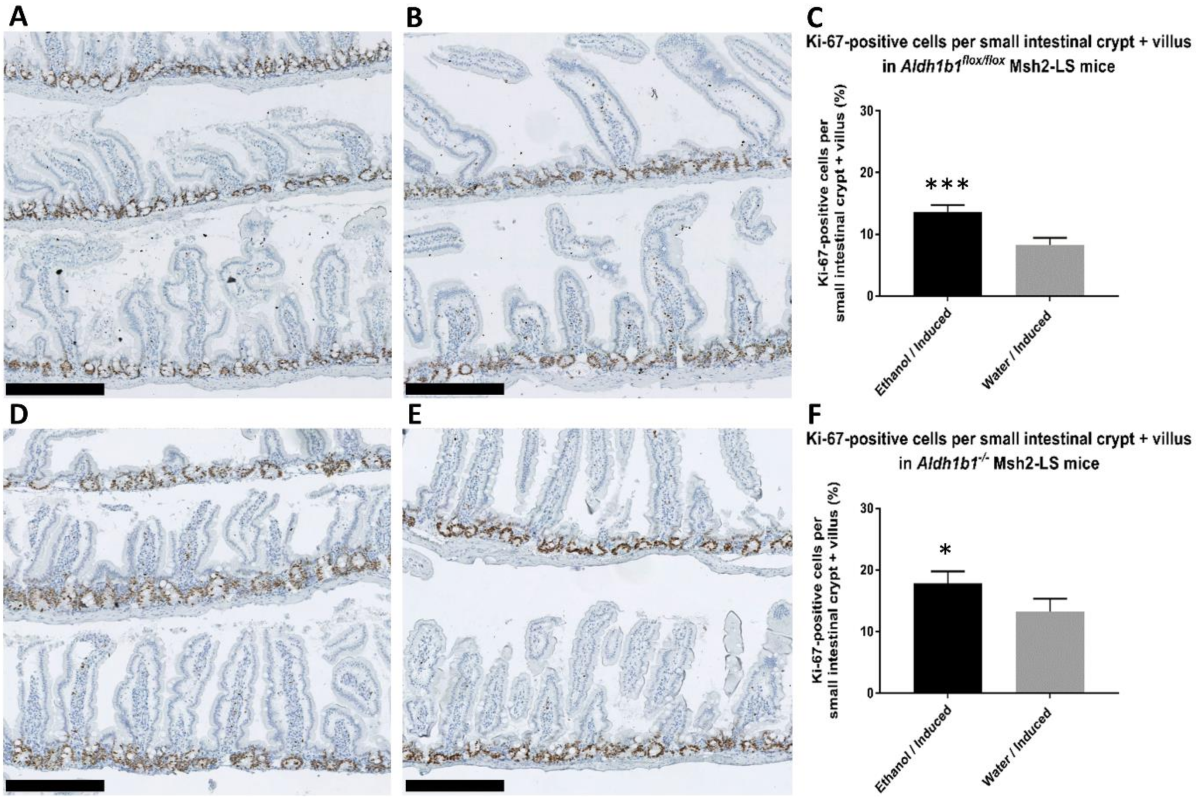

Fig. S10. Representative images of SI immunostained for Ki-67 with quantification of numbers of Ki-67-positive SI cells per crypt plus villus. Immunohistochemical analysis of Ki-67 protein expression in small intestinal mucosa of induced *Aldh1b1*$^{flox/flox}$ Msh2-LS mice treated with either 20% ethanol (A) or water (B). C) Percentage of Ki-67 protein expressing cells per small intestinal crypt plus villus in small intestinal mucosa of induced *Aldh1b1*$^{flox/flox}$ Msh2-LS mice treated with either 20% ethanol or water; unpaired two-tailed Students t-test, ***p=0.0005 vs. water. Immunohistochemical analysis of Ki-67 protein expression in small intestinal mucosa of induced *Aldh1b1*$^{-/-}$ Msh2-LS mice treated with either 20% ethanol (D) or water (E). F) Percentage of Ki-67 protein expressing cells per small intestinal crypt plus villus in small intestinal mucosa of induced *Aldh1b1*$^{-/-}$ Msh2-LS mice treated with either 20% ethanol or water; unpaired two-tailed Students t-test, *p=0.0127 vs. water. Data shown as mean±SD error bars, 30 crypts plus villi per mouse were analysed, n=6 mice from each group. Images taken at magnifications of 100X (A, B, D, E), scale bars A, B, D, E: 250µm.

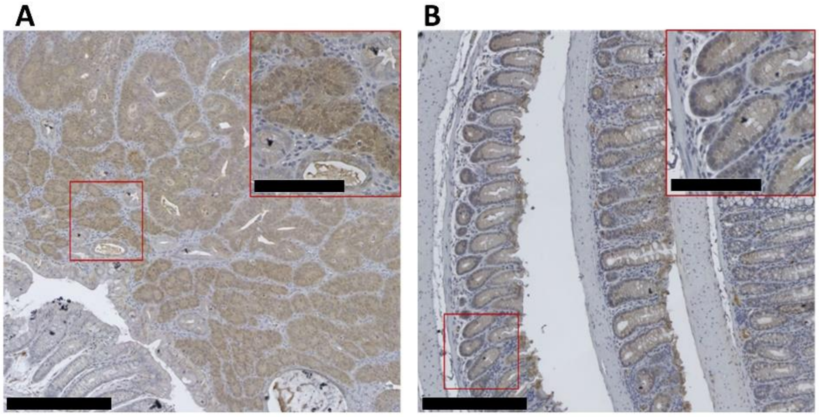

Fig. S11. Representative images of LI adenoma or LI normal mucosa immunostained for β-catenin. Immunohistochemical analysis of β-catenin protein expression and localisation in large intestinal mucosal adenoma and adjacent normal epithelium from a positive-control *Apc*-Min mouse (A), in which the increased brown nuclear β-catenin staining in variable numbers of adenoma cells in a heterogeneous pattern confirms dysregulation of Wnt signalling in this neoplastic intestinal epithelium (further magnified in the red rectangle); and from a normal control wild-type mouse (B), in which the membranous and cytoplasmic β-catenin light staining indicates normal large intestinal mucosal epithelium without activation of the Wnt signalling pathway. Images taken at magnifications of 100X (main images A and B) and 200X (inset red rectangles), scale bars A, B: 250µm (inset red rectangles in A,B: 100µm).

Disease Models & Mechanisms • Supplementary information

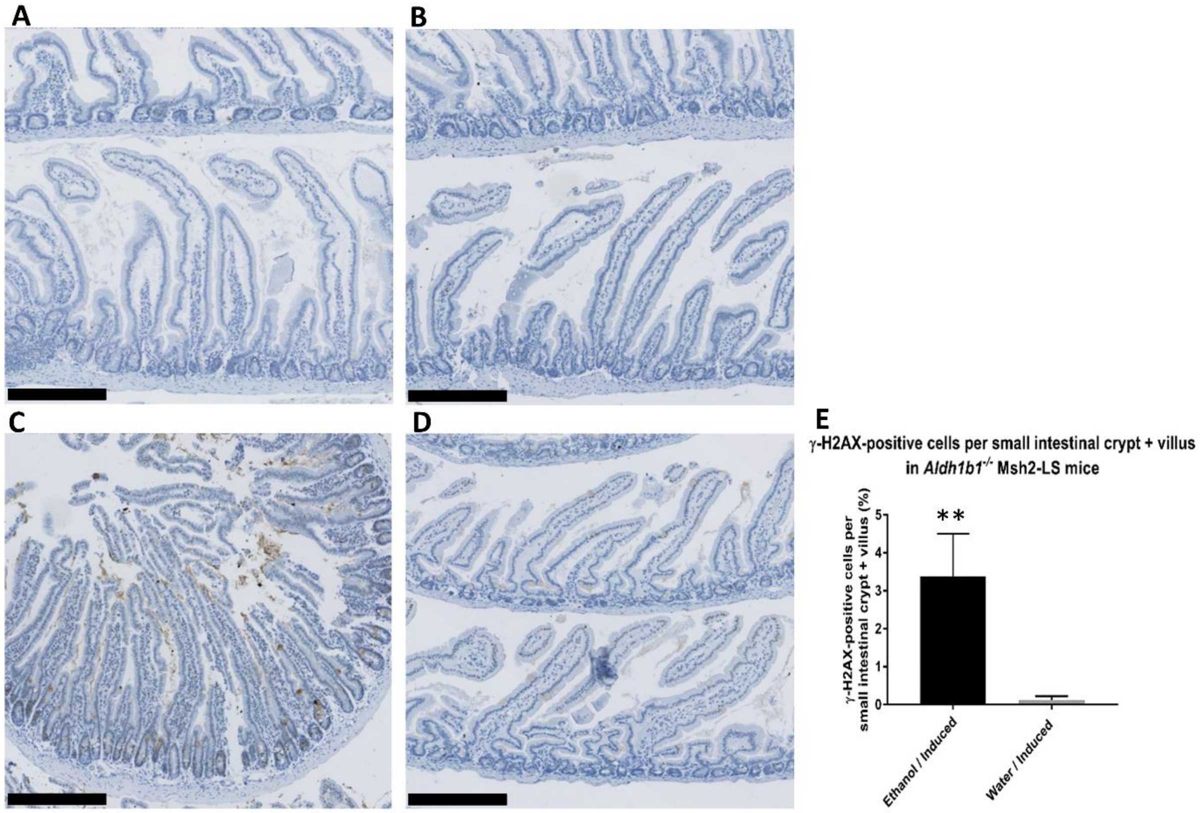

Fig. S12. Representative images of SI immunostained for γ-H2AX with quantification of γ-H2AX-positive cells per SI crypt plus villus. Immunohistochemical analysis of γ-H2AX protein expression in small intestinal mucosa of induced *Aldh1b1*$^{flox/flox}$ Msh2-LS mice treated with either 20% ethanol (A) or water (B); no γ-H2AX-positive cells were observed in either sample. Immunohistochemical analysis of γ-H2AX protein expression in small intestinal mucosa of induced *Aldh1b1*$^{-/-}$ Msh2-LS mice treated with either 20% ethanol (C) or water (D). E) Percentage of γ-H2AX-positive cells per small intestinal crypt plus villus of induced *Aldh1b1*$^{-/-}$ Msh2-LS mice treated with either 20% ethanol or water; unpaired two-tailed Students t-test, **p<0.0012 vs. water (data shown as mean±SD, 40 crypts plus villi per mouse were analysed, n=4 mice in each group). Images taken at magnifications of 100X (A, B, C, D), scale bars A, B, C, D: 250µm.

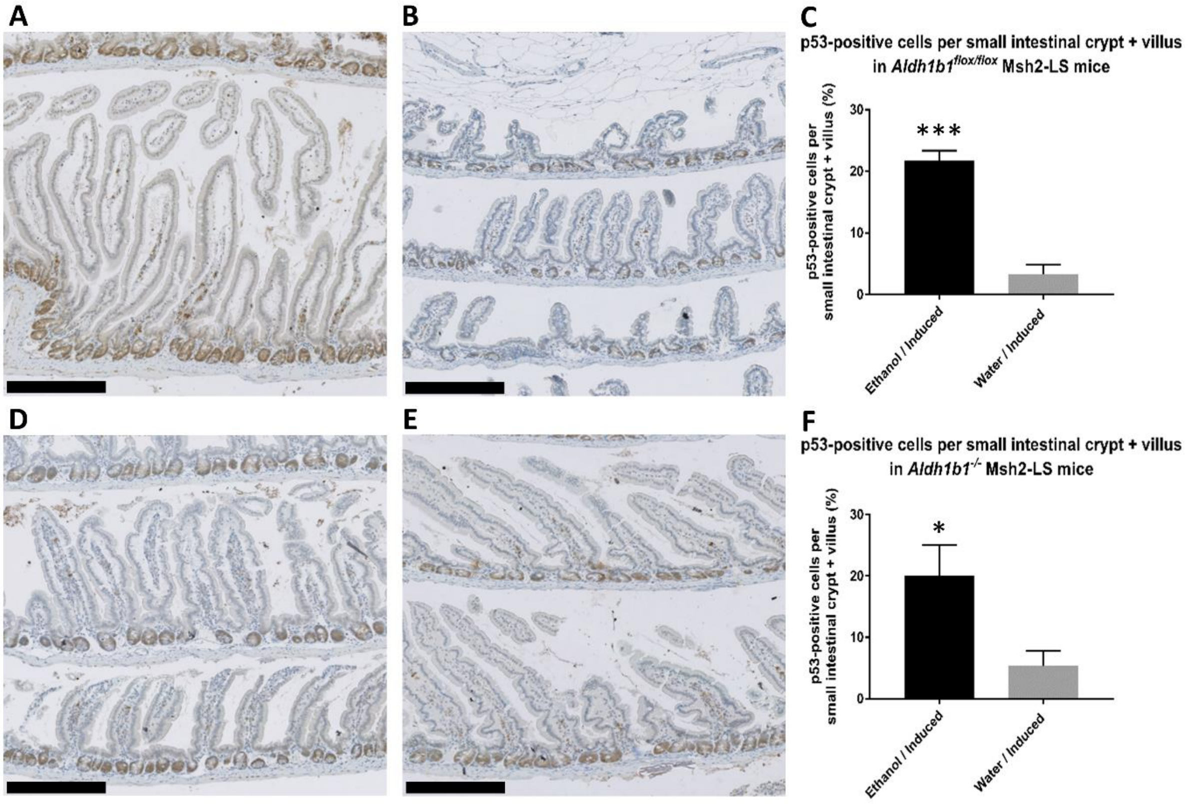

Fig. S13. Representative images of SI immunostained for p53 with quantification of p53-positive cells per SI crypt plus villus. Representative images of immunohistochemical analysis of p53 protein expression in small intestinal mucosal epithelium of induced *Aldh1b1$^{flox/flox}$* Msh2-LS mice treated with either 20% ethanol (A) or water (B). C) The percentage of positive p53-nuclear stained cells per small intestinal crypt plus villus in small intestinal mucosa of induced *Aldh1b1$^{flox/flox}$* Msh2-LS mice treated with either 20% ethanol or water; unpaired two-tailed Students t-test, ***p<0.0001 vs. water. Representative images of immunohistochemical analysis of p53 protein expression in small intestinal mucosal epithelium of induced *Aldh1b1$^{-/-}$* Msh2-LS mice treated with either 20% ethanol (D) or water (E). F) The percentage of positive p53-nuclear stained cells per small intestinal crypt plus villus in small intestinal mucosa of induced *Aldh1b1$^{-/-}$* Msh2-LS mice treated with either 20% ethanol or water; unpaired two-tailed Students t-test, *p<0.0105 vs. water. Data shown as mean±SD, 40 crypts plus villi per mouse were analysed, n=4 mice in each group. Images taken at magnifications of 100X (A, B, D, E), scale bars A, B, D, E: 250µm.

Disease Models & Mechanisms • Supplementary information
